# Development and evaluation of an intervention to increase the collection of compostable packaging from households for industrial composting

**DOI:** 10.1177/0734242X251328964

**Published:** 2025-04-21

**Authors:** Meghann Matthews, Thomas L Webb, Sarah Greenwood, Rosie Sharp, Carla Roberts-Owen, Eric Saldanha, Tom McBeth, Nicola J Buckland

**Affiliations:** 1 School of Psychology, University of Sheffield, Sheffield, UK; 2 School of Mathematical and Physical Sciences, University of Sheffield, Sheffield, UK; 3 Grantham Centre for Sustainable Futures, University of Sheffield, Sheffield, UK; 4 Hubbub, London, UK; 5 RECOUP, Peterborough, UK

**Keywords:** Compostable packaging, behaviour change intervention, waste behaviour, sustainability, capability, opportunity, motivation-behaviour model, behaviour change wheel

## Abstract

Certified compostable packaging has the potential to be a more sustainable alternative to some conventional plastics, but only if disposed of appropriately. This study developed and evaluated the effects of a behaviour change intervention on the disposal of compostable packaging in the co-mingled food and garden waste bin for industrial composting in households in the United Kingdom. The intervention targeted barriers to the appropriate disposal of compostable packaging as identified via focus groups, previous research and the capability, opportunity, motivation-behaviour model. Intervention components included front- and back-of-pack labels, tips on positioning of household bins, visual reminders to check packaging labels and which bin to use, and a bag of compost with an infographic highlighting what happens during the composting process. The intervention was delivered over 6 weeks to 120 households who were provided with compostable and non-compostable items. The results from online surveys completed at three time points (pre-intervention, post-intervention and follow-up) and weekly waste audits that assessed the weight of compostable packaging in the food and garden waste bin showed significant increases in perceived capability, opportunity and motivation to correctly dispose of compostable packaging via the food and garden waste bin over the course of the intervention and increased amounts of compostable packaging disposed of. The implications are that standardised, clear labels on industrially compostable packaging are needed to help residents to identify and appropriately dispose of this packaging. Strategies that automatically prompt which bin to use and increase residents’ awareness and motivation to appropriately dispose of compostable packaging are also recommended.

## Introduction

It is thought that approximately 350 million tonnes of plastic waste is produced globally each year, and this is projected to triple by 2060 (Statista, 2024). At a more local level, households in the United Kingdom throw away an estimated 90 billion pieces of plastic a year (Greenpeace, 2022). From this, 17% is recycled, 58% is incinerated, 14% is exported and 11% is sent to landfill. This growing accumulation of plastic waste poses a threat to natural environments and human health (WRAP, 2020), and so it is crucial that more sustainable alternatives to plastic packaging are used. Certified compostable packaging, which breaks down into water and carbon dioxide when industrially composted,^1^ offers a potentially more sustainable alternative to some conventional packaging, particularly for hard-to-recycle items, such as flexible packaging (e.g. crisp packets) and/or where packaging is contaminated by food (WRAP, 2020). Yet, the potential environmental benefits of compostable packaging depend on people correctly disposing of compostable packaging via waste streams that are collected for industrial composting. However, research indicates that people often dispose of compostable packaging via inappropriate waste streams, such as the recycling bin (e.g. Ansink et al., 2022; Closed Loop Partners’ Composting Consortium & Biodegradable Products Institute, 2023; Taufik et al., 2020) or via home composting heaps which often do not meet the requirements needed for industrial composting (Purkiss et al., 2022). This is perhaps not surprising given that few – if any – local authorities in the United Kingdom collect compostable waste; indeed, there is significant variability between local authorities in terms of what is collected, but also how (e.g. what waste streams are combined) and what the bins look like (e.g. Eida, 2024). It therefore seems likely that households will need support to ensure compostable packaging is disposed of in the appropriate bin if compostable packaging is to be collected for industrial composting.

Compostable packaging is increasingly available – with further forecasted 15–17% market increases by 2027 (Packaging World, 2023). There is also public willingness to purchase items in compostable packaging (e.g. Purkiss et al., 2022), suggesting that it is likely that compostable packaging will represent a larger share of the packaging that households encounter and need to dispose of. As such, effective household waste collection systems, whereby compostable packaging is collected from households for industrial composting, may be needed. However, the effectiveness of such collections will depend on households being able to identify compostable packaging and put it in the appropriate bin for industrial composting. When waste is put in the incorrect bin, this can result in contamination of that waste stream. Contaminated waste streams can present several challenges including increased costs due to the need of additional sorting, a decrease in the value of any recyclable materials and/or a reduction in the amount of material that can successfully recycled or appropriately disposed of (Cakanlar et al., 2024; Recycle Now, n.d.). As such, it is important to understand the barriers to households’ disposal of compostable packaging via waste streams intended for industrial composting, and where needed, develop interventions to improve the appropriate disposal of compostable packaging.

Research has started to examine potential strategies to encourage households’ appropriate disposal of compostable packaging. For example, Allison et al. (2022c) applied the behaviour change wheel (BCW; Michie et al., 2011, 2014) to design a label for compostable packaging. The idea was to help people to distinguish compostable packaging from conventional recyclable plastics – something that is often challenging, especially given that currently in the United Kingdom there is no standardised label used for compostable packaging (Allison et al., 2021a; Buckland et al., 2025; Purkiss et al., 2022; Taufik et al., 2020). Similarly, there are no standardised or evidence-based communications for households about the waste stream to dispose of compostable packaging. However, despite proposing labels for compostable packaging, to date, no research has evaluated the effect of labels on the disposal of compostable packaging in day-to-day life within households. Therefore, it remains unclear what effect labels and other behaviour change interventions which target barriers to the appropriate disposal of compostable packaging will have on households’ disposal of compostable packaging where household waste is collected for industrial composting. Consequently, the current study aims to address this gap in the literature.

## Behaviour change frameworks

**Figure 1.**
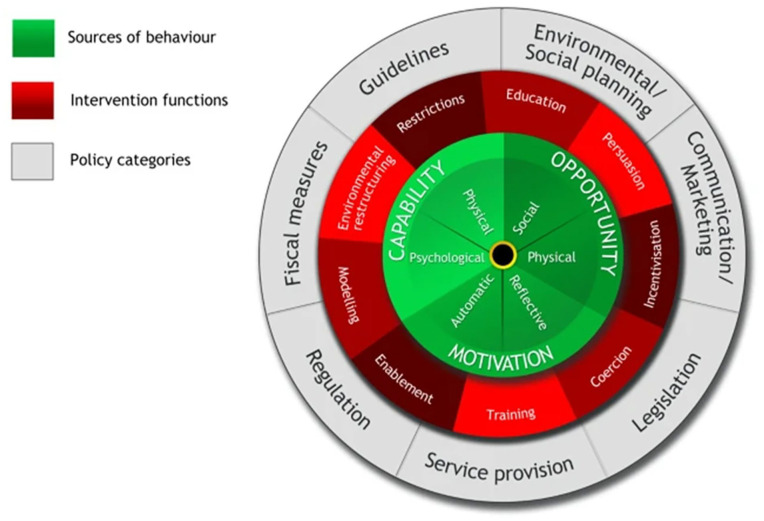
The behaviour change wheel (Michie et al., 2011, 2014).

Numerous behaviour change models that can guide the development of behaviour change interventions. One model that has been used in a range of domains is the BCW (Michie et al., 2011, 2014 see Figure 1), which provides a comprehensive and systematic framework for designing and evaluating interventions, comprising of three broad phases: (i) understanding the target behaviour and identifying what needs to change, (ii) selecting intervention options and (iii) identifying implementation options. The first stage involves identifying and specifying the target behaviour. The action, actor, context, target, time (AACTT) framework (Presseau et al., 2019) suggests that it is important to specify what behaviour is being targeted (action), who is performing the behaviour (actor), the circumstances or conditions the behaviour occurs in (context), who the behaviour is targeted at/effects (target) and when the behaviour is performed (time).

Once the target behaviour has been identified, it is important to understand what factors influence that behaviour, as these can serve as potential targets for the intervention. Here, the capability, opportunity, motivation-behaviour (COM-B) model (Michie et al., 2011, 2014) – can be used. The COM-B model suggests that for a behaviour to occur, an individual must have the capability, opportunity and the motivation to perform the targeted behaviour (see the inner hub of the BCW in Figure 1). Capability refers to an individual’s ability and capacity to enact the behaviour and consists of physical capability (e.g. skills) and psychological capability (e.g. knowledge). Opportunity refers to external factors that can make the behaviour possible, including physical (e.g. access to resources, infrastructure) and social (e.g. social norms) opportunities. Motivation refers to the individual’s drive to engage in the behaviour and can be automatic (e.g. emotions and unconscious responses) or more reflective (e.g. beliefs and intentions). Capability, opportunity and motivation can either promote and enable behaviour (where present) or be a barrier and prevent behaviour (where absent). Therefore, identifying the enablers and barriers to the behaviour can inform what needs to be targeted to enable the behaviour.

During the second stage, the BCW suggests nine potential intervention functions that link to different policy options (e.g. education, persuasion, training, restriction, environmental restructuring, see the second and outer layer in Figure 1). The third stage brings together the first and second stages to map the targeted barriers/enablers and the intervention functions onto specific behaviour change techniques (BCTs) to inform the content of the intervention and the mode of delivery (Michie et al., 2014). BCTs are the smallest parts of the intervention that are observable, replicable and have the potential to bring about behaviour change. BCTs can be described using the Behaviour Change Technique Ontology (Marques et al., 2023), which is part of the broader Behaviour Change Intervention Ontology (BCIO; Michie et al., 2021).

The BCW has been used to develop behaviour change interventions to target recycling (Allison et al., 2022b; Gainforth et al., 2016), reuse (Allison et al., 2021b) and the disposal of compostable packaging in closed-loop contexts, where compostable packaging is circulated and collected on-site (WRAP, 2020). For example, Buckland et al. (2025) developed an intervention which included distinctive packaging labels with clear disposal instructions, bin signage that visually matched the packaging labels and a motivational video and an onboarding presentation to workplace managers. Buckland et al. (2025) found that the intervention increased the amount of compostable packaging collected from compostable bins at three workplaces. Such findings, alongside a meta-analysis (Allison et al., 2022a), suggest that behaviour change interventions have the potential to improve the appropriate disposal of compostable packaging. However, closed-loop contexts offer a controlled and structured environment for the use and disposal of compostable packaging. Further research is needed to investigate whether behavioural interventions can also improve households’ appropriate disposal of compostable packaging within contexts where it is collected for industrial composting.

## The current study

The current study aimed to develop and evaluate an intervention to encourage households in the United Kingdom to appropriately dispose of compostable packaging. Specifically, households were encouraged to dispose of compostable packaging in their co-mingled food and garden waste bin (a brown coloured wheelie bin intended for both food and garden waste), during a trial period when the local authority accepted compostable packaging in these bins. Prior to the study, the local authority provided no communications about compostable packaging and did not collect compostable packaging for industrial composting. The current study had multiple specific objectives, including:

1. To explore barriers and enablers to households’ appropriate disposal of compostable packaging.
2. To apply the COM-B model and BCW to design an intervention to target the identified barriers (intervention development phase).
3. To evaluate the effects of the intervention on:
  a. Households’ perceived capability, opportunity and motivation to appropriately dispose of compostable packaging
  b. Households’ responses to an online behavioural choice task where they were asked to choose waste disposal options for compostable and non-compostable items and indicate their confidence in the waste disposal decision
  c. Households’ self-reported behaviour regarding disposal of compostable packaging
  d. The amount of compostable packaging disposed of in the food and garden waste bin (directly or indirectly via the kitchen caddy for food waste), as assessed by weekly waste audits

All outcomes were measured at three time points: pre-intervention (weeks 1 and 2), post-intervention (weeks 3 and 4) and follow-up (weeks 5 and 6). Furthermore, the acceptability of the intervention was assessed after the intervention.

It was hypothesised that participants would report significantly greater capability, opportunity and motivation to identify and correctly dispose of compostable packaging after the intervention compared to before. In the behavioural choice task, it was expected that participants would correctly put more compostable packaging in the food and garden waste bin and feel more confident in their choices after the intervention compared to before. Finally, it was expected that more compostable packaging would be disposed of in the food and garden waste bin, with less contaminating materials (e.g. non-food/garden/compostable packaging waste), after the intervention compared to before the intervention.

## Methods

The research involved two main phases. Firstly, the development phase assessed barriers and enablers to households disposing of compostable packaging via their food and garden waste bin for industrial composting. The barriers and enablers were mapped to COM-B components to identify which components to target during the intervention. The BCW (Michie et al., 2011) was then applied to guide intervention development. Secondly, the evaluation phase involved delivering the intervention and measuring beliefs specified by the COM-B model and waste behaviour. After the intervention, a sub-sample of households shared their experiences and acceptability of the intervention via two focus groups. All aspects of the research received ethical approval from the University of Sheffield ethics committee and were pre-registered on the Open Science Framework (OSF; https://osf.io/xfgwj/). The materials and datasets for each of the studies can also be found on the OSF. The content, target(s) and modes by which the intervention was delivered are reported using standardised frameworks – specifically, the BCIO (Marques et al., 2023).

## Development phase

The target group were households (BCIO: OMRSE:00000076) in a local authority in the South East of England, United Kingdom (namely, Medway Council). Previous research (e.g. Allison et al., 2022b; Buckland et al., 2025) and discussions with project partners, were used to identify the behaviours to target in the intervention. Specifically, using the AACTT framework (Presseau et al., 2019), we were interested in residents’ (actor) disposal of compostable packaging into the co-mingled food and garden waste bin (action) at the end-of-life (time) in households (context) where co-mingled food and garden waste were collected for industrial composting (target). The study focused on household disposal of compostable packaging into the food and garden waste.^2^ This was a new behaviour for residents, because prior to the study, compostable packaging was not collected from households and residents received no communications about how to dispose of compostable packaging.

To identify the main barriers to the appropriate disposal of compostable packaging, we reviewed previous research (e.g. Allison et al., 2022c; Buckland et al., 2025) and conducted 5 focus groups with 22 household residents (6 men, 16 women), aged between 31 and 72 years (*M* *=* 51.0, SD *=* 12.8 years). The details and findings of these focus groups are reported in Supplemental Material 1 and the OSF (https://osf.io/ztvku). Meetings were then held between researchers at the University of Sheffield and Hubbub to generate and refine intervention options to address the main barriers identified. The discussions were structured using the COM-B framework (e.g. intervention components targeting capability were separated from those targeting motivation) and potential options were evaluated with respect to the criteria specified by the EAST framework (Behavioural Insights Team, 2014) in which we considered whether they were easy, attractive, social and timely. Discussions were also held with project partners to gain feedback on the development of the intervention components.

Table 1 summarises the barriers that were identified (mapped to COM-B components), the intervention(s) that were developed to address each barrier, along with the functions of these interventions, specific BCTs and mode of delivery. In brief, the intervention consisted of front- and back-of-pack labels, clearly identifying compostable packaging (the back-of-pack label matched the colour of the food and garden waste bin); tips on the positioning of the bins; visual reminders to check packaging labels and use the food caddy and a bag of compost with an infographic describing the composting cycle. Supplemental Material 2 provides further information on the content of the meetings that were used to develop the intervention and additional information regarding the intervention components.

**Table 1.** Barriers to appropriately disposing of compostable packaging (categorised by COM-B component that they reflect) and associated intervention (specified in terms of the intervention component, function, behaviour change technique(s) and mode of delivery).

Barrier	COM-B component	Intervention	Intervention function	Behaviour change technique(s)	Mode of delivery
Difficulty identifying compostable packaging	Capability (psychological)	Front- and back-of pack labels on all compostable items supplied to households	Education	Cue (BCIO:007081)	Labelling mode of delivery (BCIO:011009)
Confusion regarding which bin to put compostable packaging in	Capability (psychological)	Back-of-pack label on compostable packaging which specified which bin to use	Education/enablement	Instruct on how to perform behaviour (BCIO:007058)	Labelling mode of delivery (BCIO:011009)
Storage space and/or access to bins is challenging	Opportunity (physical)	Tips on positioning bins (encouraged to use kitchen food waste caddy as an intermediary bin to collect compostable packaging and to position this in an accessible place in the kitchen) Eye mask wrap for households to put on kitchen caddies. A tag for households to attach to their food and garden waste bin	Environmental restructuring	Restructure the physical environment (BCIO:050348) BCIO:007156 (add objects to the environment) BCIO:007158 (identify self as a role model)	Printed material mode of delivery (BCIO:011005) Electronic mode of delivery (BCIO:011010)
Lack of awareness of the composting process and the resulting outputs	Motivation (reflective)	Bag of compost and infographic outlining the composting process	Education/persuasion	Increase awareness of consequences (BCIO:007062) Inform about environmental consequences (BCIO:007176)	Printed material mode of delivery (BCIO:011005) Visual informational mode of delivery (BCIO:011031)

Alongside the intervention components, 26 households (22%) received a kitchen food caddy (prior to the research, the local authority provided caddies to households as part of waste management provisions).

COM-B: capability, opportunity, motivation-behaviour; BCIO: Behaviour Change Intervention Ontology (https://www.bciontology.org/).

## Evaluation phase

### Study design

A pre–post design was used to test assess the effects of the multi-component intervention on: (i) perceived capability, opportunity and motivation to appropriately dispose of compostable packaging; (ii), responses to an online behavioural choice task designed to measure how households would dispose of different types of packaging and (iii) the amount of compostable packaging in the food and garden waste bin. The study was conducted for 6 weeks from October to November 2023. The intervention lasted 6 weeks and consisted of 3 phases: (i) a pre-intervention phase (weeks 1 and 2); (ii) a post-intervention phase (weeks 3 and 4) and (iii) a follow-up phase (weeks 5 and 6). During each phase, households (i) received a food box containing items in compostable and non-compostable packaging to ensure access to compostable packaging and (ii) completed a survey to assess putative determinants of behaviour (i.e. COM-B components). Households were asked to dispose of the compostable packaging in their co-mingled food and garden waste bin for the duration of the intervention, which would then be collected for industrial composting.^3^ To assess what material was disposed of and where, in line with the local council collections, waste audits were conducted on a weekly basis. The (second and third) food boxes were also used as the mode of delivery for the intervention components, except for the bag of compost and the accompanying infographic, were delivered in the second food box (week 3). The bag of compost and the infographic were delivered with the third food box (week 5). In week 3, participants also received an SMS message with the tips for positioning their bins.

During the 2-week pre-intervention phase, households received information informing them that they could now use the food and garden waste bin for compostable packaging (BCIO:007058; Instruct on how to perform behaviour). This information was delivered via flyer within the first food box and was designed in line with previous communications provided to households by the local authority. The multi-component intervention was then delivered from the start of the intervention phase.

### Participants and recruitment

An a priori power calculation conducted in G*Power suggested that 44 and 68 participants would provide 80% or 95% power, respectively, to detect a medium effect of the intervention on survey outcomes (Cohen’s *f*^2^ of 0.20, based on the typical effect of interventions targeting behaviours related to plastic waste, Allison et al., 2022a) using a repeated measures multivariate analysis of variance (MANOVA) at the standard 0.05 alpha error probability. As attrition poses a challenge for longitudinal research, with attrition rates of between 30% and 70% previously being reported (Gustavson et al., 2012), the current research aimed to recruit 120 households to allow up to 70% attrition while still providing >95% power.

Based on Area Classification for Output Areas (Office for National Statistics, 2021), we aimed to recruit residents from suburbanite and hard-pressed living areas as these represent 46% of the areas in the local authority within which the intervention was to be conducted. Medway Council identified four streets within the suburbanite and hard-pressed living areas where household waste could be collected for industrial composting for the intervention period (Index of Multiple Deprivation deciles, two streets: 4; two streets: 8; UK Government, 2019).^4^ Households were recruited through flyers posted through letterboxes, put on lampposts in the selected streets and via targeted local social media adverts. The flyers included a link and QR code to a screening survey to determine eligibility. Project partners and council staff also knocked on doors to encourage recruitment and answer any questions.

To participate, individuals had to: (i) be at least 18 years old, (ii) live on one of the four streets identified by the local council, (iii) be available to receive the three food boxes and put waste out on at least five of the six collection days, (iv) have access to a food and garden waste bin and (v) be contactable via text and email. The screening survey also asked residents when they preferred to be contacted, if they had a Nespresso compatible coffee machine (as these were one of the products that could potentially be included in the food boxes) and a food caddy, how many people live in the household, whether any members of the household had food allergies and whether they had a home compost heap. Households without a food caddy (26 households, 22%) were provided with one. Twelve households reported having a home compost heap (14%), but none of the households reported using their home compost heap to dispose of compostable packaging. Participants were informed that the study was a trial of food and drink packaging, with the focus on compostable packaging being concealed during recruitment to avoid potential bias.

The screening survey was completed by 157 households, 120 of which were eligible and confirmed participation in the intervention. One household withdrew before the first survey was circulated; thus, the final sample consisted of 119 households.

### Materials

#### Food boxes

Three food boxes were delivered to households, each containing at least 13 items (14 items for the households who had compatible coffee machines) including tea bags, chocolate bars, pumpkin seeds, crisps, fresh fruit, vegetables, twist wrapped sweets, chocolate raisins and compostable carrier bags that the households were encouraged to use to line their food caddies. The items were provided by a number of companies. Four items were in non-compostable packaging (apple bag, popcorn, crisps and chocolate raisins), while all other items had at least one element that could be composted (e.g. compostable tea bags in a recyclable box). Each food box contained the same items, along with leaflets containing information about the research (e.g. delivery dates, researcher contact details) and the intervention (e.g. what to put in each bin and tips on bin positioning).

#### Surveys

Households were asked to complete three surveys: pre-intervention (survey 1), post-intervention (survey 2) and follow-up (survey 3). One week after the delivery of the first and second food boxes, participants received the links to the pre-intervention and post-intervention surveys, respectively, via SMS. The final, follow-up survey was shared 2 weeks after the delivery of the final food box to reduce the influence of the questions on the final waste audits. Each survey was open for 1 week. All of the surveys were hosted on Qualtrics (Provo, UT, United States). Households received shopping vouchers for completing the surveys: £10 for survey 1, £10 for survey 2 and £15 for survey 3.

### Measures

#### Survey measures

For concision, only the key outcomes of interest are reported. Further information about all of the measures included in the surveys is in Supplemental Material 3, and the full measures are reported on OSF. The following measures were included in both pre- and post-intervention surveys:

##### Perceived capability, opportunity and motivation

Participants’ beliefs about their capability, opportunity and motivation to appropriately dispose of compostable packaging were measured using 27 items based on previous research (Buckland et al., 2025). Participants were asked to rate the extent to which they agreed with statements referring to each COM-B component on a 5-point scale (1 = strongly disagree, 5 = strongly agree). Supplemental Table S2 provides the individual items and internal reliability for each subscale. The items were displayed in a randomised order. Scale scores were created for each COM-B component by averaging responses to the relevant items. Higher scores indicate greater perceived capability, opportunity and/or motivation.

##### Self-reported disposal of compostable packaging

Participants were asked ‘How frequently has your household put compostable packaging in the food and garden waste bin over the last week?’ and were asked to respond on a 6-point scale (1 = never, 6 = always). Participants were also asked whether the type of compostable packaging (e.g. tea bag, wrapper) influenced whether it goes into the food and garden waste bin and to explain their answer. Participants indicated whether they had a composter / compost heap in their garden, and if there is a strong culture in their local community to home compost. Additionally, participants were asked how they dispose of compostable packaging at home (e.g. whether they use a food caddy, whether it goes directly into the food and garden waste bin, whether it goes into the general waste bin).

##### Online behavioural choice task

Participants were presented with 24 images of items in either compostable (14 items) or non-compostable packaging (10 items). Images depicted all the items in the food boxes and additional items in compostable packaging and non-compostable packaging (e.g. a milk bottle and tinned food). For each image, participants were asked to select which bin they would put the packaging in (e.g. the general waste bin, the food and garden waste bin, blue recycling bag (paper/card recycling), white recycling bag (plastic recycling), or other). The options aligned with the waste streams provided by the local council waste collection at the time of the research. For every trial that participants selected the correct disposal method (i.e. selected to put compostable packaging in the food and garden waste bin), they scored 1 and for every trial they selected an incorrect method of disposal, they scored 0. Higher scores therefore reflect more appropriate disposal of packaging. For each trial, participants were asked to rate how confident they were in their choice of bin (1 = not confident at all, 10 = very confident). These ratings were averaged.

##### Attention checks

Three questions were included to ensure participants were paying attention (e.g. ‘Just to check you are paying attention, please click “strongly disagree”’). Participants who incorrectly answered all the attention check questions were excluded from analyses (*n* = 2).

The following questions were included in the final survey (only):

##### Awareness of the intervention components

An image of each intervention component was displayed and participants were asked whether they had noticed each component (yes, no, unsure).

##### Acceptability of the intervention components

A modified version of the theoretical framework of acceptability (TFA; Sekhon et al., 2018) was used. For each intervention component that participants reported noticing, they were asked questions relating to the following seven constructs: affective attitude, burden, ethicality, intervention coherence, self-confidence, opportunity cost and perceived effectiveness. We also assessed general acceptability. Participants were asked to respond on a 5-point scale. The majority of the response scales were agreement scales (1 = strongly disagree, 5 = strongly agree), but other response options reflected the construct (e.g. strongly disliked – strongly liked; no effort at all – huge effort; very unfair – very fair; very unconfident – very confident; completely unacceptable – completely acceptable).

#### Waste measures

During the trial, the food and garden waste bins and the dry mixed recycling bags of the participating households were collected weekly on the local council’s usual waste collection day, but by a different company. The collection crew recorded whether the waste was presented for collection, any contamination (i.e. waste meant for other waste streams) and whether the bin tag from the intervention had been attached to the food and garden waste bin. Each week, the waste from the food and garden waste bins was weighed by an auditor at a facility following standard procedures. The compostable packaging was weighed, as was any contamination in these bins. At each time point (pre-intervention, post-intervention and follow-up), the waste from all participating households was combined (but kept as separate waste streams). As such, the data reflected the overall weight (in grams) and percentage of waste in the food and garden waste bins that was (i) compostable packaging, (ii) garden waste, (iii) food waste and (iv) contaminating materials at each time point for all households combined. In week 1, an error with the waste collections resulted in approximately half of the households’ food and garden waste being co-mingled with other waste streams. The data were treated as missing and, in a deviation from the pre-registered protocol, the total waste (grams) was divided by the number of households from which the waste was collected to provide a proportion of waste by household.

## Data analysis plan

A repeated measures MANOVA was conducted with the six COM-B subcomponents as dependent variables and time (pre-intervention, post-intervention, follow-up) as the within-subjects factor.^5^ A number of one-way repeated measures ANOVAs were conducted to examine: (i) whether there were any changes in participant’s self-reported measures of disposal of compostable packaging in the food and garden waste bin over the intervention, (ii) whether there were changes in participants’ behaviour regarding which bin to dispose of compostable packaging in over the intervention and (iii) whether there were any changes in how confident participants were in their choices. Where relevant, partial eta squared (partial η^2^) is reported for effect sizes and interpreted as 0.01 = small; 0.06 = medium; 0.14 = large effect. Descriptive statistics were examined for the waste audits, in line with previous research (e.g. Woodard et al., 2001). Similarly, descriptive statistics were examined for the questions pertaining to the acceptability of the intervention.

## Results

### Survey responses

About 112 (94%) of the 119 participating households completed the pre-intervention survey. Supplemental Table S3 shows the characteristics of participants. Most of the participants were women, married, identified as White, were not University educated and were employed or retired. In total, 100 (84%) completed the post-intervention survey and 96 (81%) completed the follow-up survey. Eighty-nine participants (75%) completed all three of the surveys. Of these 89 participants, 2 participants failed attention checks and 1 participant provided suspicious data (i.e. provided the same response to every item). Therefore, the following analyses are based on the 86 complete responses (72%) across the 3 surveys. This number met the required minimum sample size as per power calculations. Before the analyses were conducted, the assumptions made by the statistical analyses were checked and further information can be found in Supplemental Material 4.

#### Changes in perceived capability, opportunity and motivation

Table 2 shows the descriptive statistics for perceived psychological and physical capability, physical and social opportunity and reflective and automatic motivation to identify and appropriately dispose of compostable packaging over the intervention. A repeated measures MANOVA was conducted with the six COM-B components as dependent variables and time (pre-intervention, post-intervention, follow-up) as the within-subjects factor. There were statistically significant changes in the six COM-B components over the intervention, *F*(12, 62) = 3.94, *p* < 0.001; Wilks’ Λ = 0.57; partial η^2^ = 0.43.

**Table 2.** Means (and standard deviations) for capability, opportunity and motivation for disposing of compostable packaging in the co-mingled food and garden waste bin at pre-intervention, post-intervention and follow-up (*n* = 74).

COM-B component	Pre-intervention	Post-intervention	Follow-up
Psychological capability	3.80 (0.63)	4.01 (0.59)	4.12 (0.67)
Physical capability	4.25 (0.90)	4.36 (0.84)	4.36 (0.96)
Physical opportunity	3.56 (0.66)	3.69 (0.71)	3.88 (0.78)
Social opportunity	3.03 (0.80)	3.01 (0.51)	3.06 (0.49)
Reflective motivation	3.83 (0.57)	4.13 (0.57)	4.20 (0.55)
Automatic motivation	3.78 (0.84)	3.96 (0.72)	4.00 (0.71)

Possible scores ranged from 1 to 5. Higher scores indicated greater capability, opportunity and motivation.

Univariate ANOVAs and Bonferroni-corrected follow-up analyses (see Supplemental Table S6) showed that there were significant increases in perceived psychological capability, physical opportunity, reflective motivation and automatic motivation between pre-intervention and follow-up. Additionally, there were significant increases between pre-intervention and post-intervention for psychological capability and reflective motivation and between post-intervention and follow-up for physical opportunity. There were no significant changes in physical capability or social opportunity between any time points.

#### Self-reported disposal of compostable packaging in the food and garden waste bin

A one-way ANOVA revealed that the intervention had a significant effect on the amount of compostable packaging households reported disposing of, *F*(1.625, 138.112) = 15.01, *p* < 0.001; partial η^2^ = 0.150. Bonferroni-corrected follow-up analyses showed that there was a significant increase in the amount of compostable packaging households reported disposing of between the pre-intervention phase (*M* = 3.99, SD = 1.83) and post-intervention phase (*M* = 4.63, SD = 1.34; mean difference = 0.64, 95% CI [0.179, 1.10], *p* = 0.003) and between the pre-intervention and follow-up phase (*M* = 4.87, SD = 1.29; mean difference = 0.88, 95% CI [0.439, 1.329], *p* < 0.001). There was no significant difference between the post-intervention and follow-up phase (mean difference = 0.24, 95% CI [−0.050, 0.538], *p* = 0.137).

#### Online behavioural choice task

A one-way ANOVA revealed a significant effect of time on the number of trials in which participants correctly selected the food and garden waste bin as a way of disposing of compostable packaging *F*(1.491, 126.776) = 51.35, *p* < 0.001; partial η^2^ = 0.377. Bonferroni-corrected follow-up analyses highlighted that there was a significant increase in the number of trials (out of 14) participants correctly disposed of compostable packaging between pre-intervention (*M* = 7.57, SD = 3.17) and post-intervention (*M* = 9.95, SD = 2.79; mean difference = 2.38, 95% CI [1.565, 3.203], *p* < 0.001), and between the pre-intervention phase and the follow-up phase (*M* = 10.34, SD = 2.76; mean difference = 2.77, 95% CI [1.944, 3.591], *p* < 0.001). There was no significant difference between the post-intervention and the follow-up phase (mean difference = 0.38, 95% CI [−0.082, 0.850], *p* = 0.143).

A one-way ANOVA was also conducted to examine whether there were any changes in how confident participants were in their choices. The effect of time was statistically significant, *F*(1.773, 147.181) = 20.08, *p* < 0.001; partial η^2^ = 0.195. Bonferroni-corrected follow-up analyses highlighted significant increases in self-reported confidence in choices between the pre-intervention (*M* = 7.92, SD = 1.38) and post-intervention (*M* = 8.46, SD = 1.23; mean difference = 0.54, 95% CI [0.218, 0.859], *p* < 0.001) and between the pre-intervention and follow-up phase (*M* = 8.70, SD = 1.13; mean difference = 0.77, 95% CI [0.431, 1.113], *p* < 0.001). There was no significant difference between the post-intervention and follow-up phase (mean difference = 0.23, 95% CI [−0.013, 0.481], *p* = 0.069).

Taken together, the data suggest that over the intervention, participants correctly selected to put more compostable packaging in the food and garden waste bin and their confidence in their choices increased.

### Waste audits

**Figure 2.**
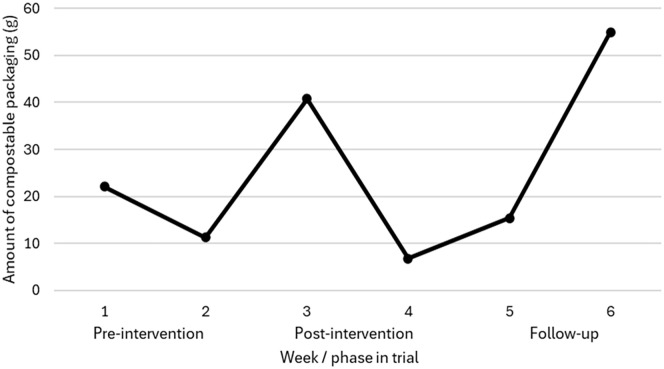
Amount of compostable packaging (grams) disposed of in the food and garden waste bin adjusted for the number of households whose food and garden waste bins were assessed each week. Weeks 1 and 2 = pre-intervention phase, weeks 3 and 4 = post-intervention phase and weeks 5 and 6 = follow-up phase. In week 1, 73 households put their food and garden waste out for kerbside collection; however, only the waste from 33 households could be analysed due to a collection error whereby food and garden waste was co-mingled with other waste streams for the other households.

With the exception of week 1 (*n* = 33), food and garden waste was collected from between 60 and 77 of the 119 households who took part in the intervention each week (see Supplemental Table S8). Figure 2 shows the amount of compostable packaging collected from food and garden waste bins adjusted for the number of households whose waste was assessed each week. Between pre-intervention (average of weeks 1 and 2) and post-intervention (average of weeks 3 and 4), the amount of compostable packaging collected from the food and garden waste bin increased by 40% (average increase of 6.8 g adjusted for the number of households whose waste was assessed). Between pre-intervention (average of weeks 1 and 2) and follow-up (average of weeks 5 and 6), the amount of compostable packaging collected from the food and garden waste bin increased by 110% (average increase of 18.5 g adjusted for the number of households whose waste was assessed). Overall, comparing the average amount of waste collected at pre-intervention (weeks 1 and 2) with after the intervention (average of weeks 3 to 6), there was a 75% increase (average increase of 12.6 g) in the amount of compostable packaging appropriately being disposed of in the food and garden waste bin.

There was also a small reduction in the percentage of contaminating materials (e.g. paper, textiles, glass) in the food and garden waste bins (where residents were directed to put their compostable material)) following the intervention (see Supplemental Table S8). Pre-intervention (average of weeks 1 and 2), 8% of the waste in the food and garden waste bin comprised of contaminating materials. This reduced to 6% at post-intervention (average of weeks 3 and 4) and 5.5% at follow-up (average of weeks 5 and 6). There was also an increase in the amount of food waste disposed of in the food and garden waste bin after the intervention compared to before. At post-intervention (average of weeks 3 and 4), food waste increased by 137% compared to pre-intervention (average of weeks 1 and 2). At follow-up, food waste increased by 123% compared to pre-intervention. Overall, comparing an average of baseline weeks (1 and 2) to after the intervention (weeks 3–6), food waste increased by 130% (see Supplemental Table S8). However, as Halloween fell in week 4, seasonal pumpkin waste may have affected the amount of food waste collected. Taken together, there was evidence that prompting residents to put compostable waste in the food and garden waste bins did not increase (and may even decrease) the amount of contaminating materials in these bins.

### Acceptability of the intervention

Table 3 shows the descriptive statistics for each of the constructs specified by the TFA (Sekhon et al., 2018). Participants’ responses suggested that the front-of-pack labels helped them to (i) identify the packaging as compostable and (ii) put the compostable packaging in the food and garden waste bin. The composting infographic seemed to improve understanding of what happens to compostable packaging. Participants also thought the front-of-pack messages were the clearest intervention component for helping others to dispose of the packaging and the component that they felt most confident using. Participants liked being able to put the compostable packaging in the food and garden waste bin, largely agreed that being able to do this had moral/ethical consequences and thought this required the least amount of additional effort. In line with the findings from the questionnaire assessing acceptability, participants in the post-intervention focus groups discussed how the labels on the packaging helped them to identify compostable packaging and the appropriate disposal (e.g. ‘Having it on the front was really helpful because-, and I don’t know whether that would be-, it would influence people to actually go, “Oh, okay, we’ll buy that because that’s got compostable packaging”’.). Participants also mentioned that the back-of-pack labels provided clear disposal instructions that made it easier to know what to do with the packaging.

**Table 3.** Means (and standard deviations) for each of the theoretical framework of acceptability constructs assessed in the survey for each of the intervention components (*n* = 86).

TFA construct	Intervention component							
	Using food and garden waste bin for compostable packaging	Back of pack labels	Front of pack labels	Tips to position bins	Bag of compost	Composting infographic	Food caddy wrap	Bin tag
Number of participants (%) who noticed the intervention component	55 (64)	64 (74)	65 (75)	69 (80)	79 (92)	54 (63)	79 (92)	75 (87)
Perceived effectiveness to identify compostable packaging	NA	4.56 (0.53)	4.62 (0.55)	3.54 (1.08)	2.92 (0.92)	3.52 (1.04)	2.85 (1.05)	3.2 (1.04)
Perceived effectiveness for putting compostable packaging in the food and garden waste bin	NA	4.52 (0.64)	4.55 (0.66)	3.67 (1.08)	2.86 (0.92)	3.96 (0.89)	2.76 (1.08)	3.09 (1.12)
Perceived effectiveness for understanding of what happens to compostable packaging	3.95 (0.87)	3.56 (1.05)	3.45 (1.02)	3.55 (1.05)	3.51 (1.02)	4.17 (0.75)	2.68 (0.94)	3.72 (2.35)
Affective attitude								
Did you like the [intervention component]?	4.49 (0.60)	4.36 (0.65)	4.45 (0.59)	3.83 (0.75)	4.20 (0.77)	4.13 (0.72)	3.03 (0.99)	3.24 (1.00)
Ethicality								
There are moral or ethical consequences to the [intervention component]	3.62 (1.18)	3.41 (1.09)	3.26 (1.05)	3.23 (0.93)	3.18 (1.00)	3.43 (1.16)	2.97 (0.77)	2.92 (0.91)
Intervention coherence								
It is clear how the [intervention component] will help people to dispose of compostable packaging	4.38 (0.73)	4.34 (0.80)	4.43 (0.59)	3.91 (0.77)	3.32 (1.07)	4.06 (0.69)	3.20 (1.05)	3.33 (1.06)
Self-efficacy								
How confident did you feel using [intervention component]?	4.42 (0.79)	4.36 (0.68)	4.48 (0.66)	4.01 (0.72)	3.72 (0.86)	4.13 (0.83)	3.33 (0.81)	3.63 (0.88)
Acceptability								
How acceptable did you find using [intervention component]?	4.45 (0.83)	4.56 (0.56)	4.52 (0.59)	4.01 (0.72)	3.84 (0.87)	4.26 (0.73)	3.24 (0.85)	3.56 (1.02)
Burden								
To what extent did using the [intervention component] require any additional effort on your part?	1.42 (0.60)	1.70 (0.97)	1.45 (0.66)	1.70 (0.79)	2.09 (0.91)	1.69 (0.84)	2.29 (0.89)	1.80 (0.90)
Opportunity cost								
Using the [intervention component] interfered with my other priorities	1.45 (0.72)	1.44 (0.64)	1.58 (0.85)	1.75 (0.86)	2.04 (0.98)	1.61 (0.81)	2.30 (0.95)	1.87 (0.96)

All statements were rated on a 5-point scale where higher scores reflect more positive responses for all statements except those reflecting burden and opportunity cost. Lower scores indicate positive responses on these items.

NA indicates where the questions were not included as they were not thought to be relevant to the intervention component.

TFA: theoretical framework of acceptability.

## Discussion

The present research designed, implemented and evaluated an intervention to improve the disposal of compostable packaging in households, as part of a trial with a local authority of household collection of compostable packaging for industrial composting. The BCW (Michie et al., 2011) was used to design an intervention that targeted issues related to capability, opportunity and motivation identified in focus groups and existing research as barriers to the appropriate disposal of compostable packaging in households. The evaluation suggested that the intervention was effective in the sense that, after the intervention (compared to before), participants reported feeling more motivated, capable and perceiving greater opportunity to dispose of compostable packaging in the food and garden waste bin for industrial composting. Participants were also better able to decide which bin to use for various packaging items (as measured by an online choice task) and the self-report measures and waste audits identified increases in the amount of compostable packaging appropriately disposed of in the food and garden waste bin. In the pre-intervention survey, participants typically reported putting compostable packaging in the food and garden waste bin ‘about half of the time’, whereas in the follow-up survey participants reported putting it in the food and garden waste bin ‘most of the time’. Compared to pre-intervention, the waste audits showed a 75% increase in the amount of compostable packaging collected in the food and garden waste bin after the intervention (average of weeks 3–6). This roughly translates to an additional five compostable packaging items being disposed of correctly after the intervention.^6^ Therefore, taken together, the findings show that a behaviour change intervention can support households’ disposal of compostable packaging into the co-mingled food and garden waste bin collected for industrial composting.

The current findings align with Allison et al. (2022a) meta-analysis which suggests that behaviour change interventions can have a medium-to-large effect on waste behaviours and extend this to the disposal of compostable waste (none of the studies reviewed by Allison et al. (2022a), focused on this behaviour). The findings also extend previous work by Buckland et al. (2025), which looked at the effects of a behaviour change intervention in a closed-loop context, to the disposal of compostable packaging in a household context. Additionally, the current work supports the notion that a number of different strategies may need to be employed to encourage environmental behaviours, such as the appropriate disposal of compostable packaging (Barr & Gilg, 2005).

In terms of people’s beliefs about the disposal of compostable packaging, participants reported feeling more capable, having greater opportunity and being more motivated to appropriately dispose of compostable packaging at follow-up compared to before the intervention. Given that the COM-B model (Michie et al., 2011) suggests that capability, opportunity and motivation are crucial determinants of behaviour and the intervention components specifically targeted these beliefs, the changes in beliefs go some way towards explaining how the intervention influenced behaviour. Increases in capability related specifically to psychological capability, suggesting that participants had more knowledge about how to identify and appropriately dispose of compostable packaging after the intervention, compared to before. Increases in opportunity related specifically to increases in physical opportunity (not social opportunity), suggesting that the physical environment (e.g. use of the food caddy to store compostable packaging before disposing via/or directly into the food and garden waste bin) afforded residents the opportunity to identify and appropriately dispose of compostable packaging. However, the intervention did not shift social norms with respect to disposal of compostable packaging (e.g. participants’ beliefs about what neighbours, family and friends do). Increases in motivation reflected changes in both automatic and reflective motivation. This suggests that the residents understanding of the composting process, the resulting outputs and the potential environmental benefits improved (i.e. there were changes in reflective motivation) and appropriate disposal of compostable packaging became more habitual over the intervention (i.e. participants reported that it was ‘something that I do without thinking’, which is a key indicator of habits, Verplanken & Orbell, 2003).

These findings differ from those of Buckland et al. (2025) who reported no significant changes in capability, opportunity and/or motivation as a function of their intervention designed to increase the collection of compostable material in closed-loop sites. A potential explanation for this difference is that disposing of compostable packaging in the food and garden waste bin was a new behaviour for participants in the present research (the local authority did not collect compostable waste prior to the intervention) and the intervention was more explicit (i.e. households had to sign-up to be involved) so they may have been more aware of any changes. In contrast, in the closed-loop context (Buckland et al., 2025), the employees were already being asked to engage with this behaviour (i.e. put their compostable packaging in a specific bin) and the cues may have operated without the need for conscious deliberation, thus not changing their self-reported beliefs about capability, opportunity and motivation. Additionally, participants in the present research may have taken more active notice of waste processes within their households compared to waste disposal interventions delivered in public settings.

Taken together, as the environmental benefits of compostable packaging can depend on appropriate disposal, research understanding whether and how people do this and how this behaviour can be promoted is important and has a number of practical implications, including for policy. For example, there are currently no requirements in the United Kingdom for standardised labelling or packaging for compostable packaging. However, current and previous research (e.g. Buckland et al., 2025; Taufik et al., 2020) suggest difficulty identifying the packaging as compostable as a barrier to its appropriate disposal. In the current study, both front- and back-of packaging labels were used and rated as acceptable by participants. Therefore, we suggest that salient front-of-pack labels should clearly indicate that packaging is compostable, and the back-of-pack labels should provide standardised, clear and actionable instructions on how to dispose of the packaging. Using a distinctive and consistent colour scheme for labels, other communications and the corresponding bin use (e.g. in this study, the colour brown was consistent for the label and food and garden waste bin) can encourage the identification of compostable packaging and a clear association with the appropriate bin to use for disposal. However, there are a number of challenges of printing coloured labels. For example, there are practicalities regarding the number of colours that can typically be printed and, due to variations in waste collection systems across the United Kingdom, matching the colour of labels to different coloured bins may be difficult. Further challenges may be posed by the introduction of mandatory labelling requirements outlining whether packaging can be recycled, which is regulated by the government (e.g. Extended Producer Responsibility for Packaging), relating to timelines and the cost to businesses. Consequently, logistical challenges and government regulations regarding on-pack-labelling need to be taken into consideration.

The current findings also suggest that using motivational and relatable strategies (e.g. providing a bag of compost) help households understand what composting is and the associated outcomes. Providing information about what composting is, how it works and what the outcome is can help to educate people so that they have the knowledge and understanding of what composting is and why it is important to engage in this. In turn, this can encourage and motivate them to engage with the behaviour. Motivational strategies such as providing compost could be provided to households by a local authority if implementing a new scheme for collecting compostable material, alongside providing the opportunity to appropriately dispose of compostable packaging from home (i.e. kerbside collection).

### Strengths, limitations and future directions

The current research has several strengths. For example, waste audits were used to evaluate the outcomes of an intervention in a real-world context. The intervention targeted barriers identified by users that were coded according to theoretical frameworks for understanding behaviour (namely, the COM-B model, Michie et al., 2011), and we examined whether the intervention was considered acceptable by participants. Considering the acceptability of an intervention is important as the effectiveness of an intervention is likely to be affected by the perceived acceptability (Diepeveen et al., 2013; Sekhon et al., 2018). Additional strengths of the research include that an interdisciplinary team conducted the research, involving both academic and applied stakeholders which helped to develop and deliver an intervention that is translatable to the waste collection system and future waste collection policies.

That said, the findings, implications and recommendations from the current research should be considered in the context of some limitations. For example, while the pre–post design allows comparisons between beliefs, behaviour and outcomes before and after the intervention, due to the logistics of collecting the waste and conducting the waste analyses, it was not possible to randomise and test the effects of each specific intervention component or to compare the intervention to a control condition. As the intervention included several BCTs (Table 1), it is possible that some components were more effective than others and/or that a more parsimonious intervention would be equally effective. The lack of a control condition also opens the possibility that the effects were attributable to variables other than the intervention. Future research should consider using factorial designs to isolate the effects of specific BCTs.

There were also some limitations to the waste audits. For example, Halloween fell during week 4 of the intervention, so this week and the following weeks included some additional food waste (e.g. from pumpkins) that would be a seasonal spike. Furthermore, as seen in Figure 2, there were considerable increases in the amount of compostable packaging in week 6. This could be due to participants using the remainder of their items and disposing of the associated packaging before the study ended. However, while these issues may have increased variability in the waste audits, they likely reflect the natural variations in waste observed in real-world contexts, and so the fact that the waste audits were conducted and seemed sensitive to changes as a function of the intervention could be considered a strength of the research.

Households were provided with compostable packaging throughout the intervention to ensure that they had access to compostable packaging to dispose of. Therefore, it is likely that these households had more compostable packaging during the intervention period than other households in the United Kingdom. Thus, the amount of compostable packaging identified in the waste audits may not accurately reflect the typical amount of compostable packaging currently being disposed of by households. That said, the increase in the amount of compostable packaging after the intervention was implemented is promising and suggests that the intervention was effective. Future research may include additional waste audits at later time-points (e.g. 2-month follow-up) to examine the longer-term effects of the intervention on appropriate disposal of compostable packaging, particularly once households have finished using the products supplied to them as part of the intervention and potentially start buying products themselves in compostable packaging. Also, while the study recruited from a community sample and targeted hard-pressed areas, the sample was mostly White, employed or retired individuals who live in houses. It will be valuable for future research to conduct similar research in more underserved communities that are often underrepresented in research.

Furthermore, most local authorities do not currently accept compostable packaging in their food and/or garden waste collections. This is due to concerns regarding contamination and as food waste is typically sent to anaerobic digestion which cannot accept compostable packaging. Therefore, while the findings from the current study are promising in terms of improving people’s behaviours regarding the disposal of compostable packaging – provided packaging is clearly labelled and appropriate communications are delivered – there are wider operational considerations that affect how and whether compostable packaging can or should be collected from households. Finally, compostable packaging is still a single-use form of packaging and as such is lower in the waste hierarchy than other waste behaviours such as reuse and reducing consumption (Department for Environment Food and Rural Affairs [DEFRA], 2011). As such, it is recognised that compostable packaging will likely have a small (but still important) role in sustainable waste systems (Environment, Food and Rural Affairs Committee, 2022).

Finally, this research only focused on one local authority within the United Kingdom, which may question generalisability of the current findings to a more international audience. However, other research has identified issues regarding the disposal of compostable packaging in other regions, such as the United States (e.g. Closed Loop Partners’ Composting Consortium & Biodegradable Products Institute, 2023; Morris et al., 2024) and Germany (e.g. Taufik et al., 2020). Thus, while the present research was conducted in a UK context, the findings offer insight into why people may not appropriately dispose of compostable packaging and barriers relating to people’s capability, opportunity and motivation. Future research could examine whether the intervention is also effective in other contexts.

## Conclusion

This research provides evidence that a theory-driven behaviour change intervention can change people’s beliefs about – and their actual – ability to identify and dispose of compostable packaging and increase the amount of compostable packaging that is collected from households. This is important as compostable packaging – and in turn compostable waste – is likely to become more widely available. Based on these findings, it is recommended that packaging producers use standardised on-pack labels that clearly identify the packaging as compostable and provide clear instructions on the appropriate disposal of compostable packaging. It is also recommended that packaging labels and communications visually correspond with communications and associated bins; for example, using a consistent colour scheme to support the automatic disposal of compostable packaging into the appropriate bin. Communications that highlight the composting process may also motivate the appropriate disposal of compostable packaging. Taken together, the present findings indicate that households are able to recognise and dispose of compostable packaging effectively if they are sufficiently informed, motivated and have the opportunity to compost packaging.

## Supplemental Material

sj-docx-1-wmr-10.1177_0734242X251328964 – Supplemental material for Development and evaluation of an intervention to increase the collection of compostable packaging from households for industrial composting Supplemental material, sj-docx-1-wmr-10.1177_0734242X251328964 for Development and evaluation of an intervention to increase the collection of compostable packaging from households for industrial composting by Meghann Matthews, Thomas L Webb, Sarah Greenwood, Rosie Sharp, Carla Roberts-Owen, Eric Saldanha, Tom McBeth and Nicola J Buckland in Waste Management & Research

sj-docx-2-wmr-10.1177_0734242X251328964 – Supplemental material for Development and evaluation of an intervention to increase the collection of compostable packaging from households for industrial composting Supplemental material, sj-docx-2-wmr-10.1177_0734242X251328964 for Development and evaluation of an intervention to increase the collection of compostable packaging from households for industrial composting by Meghann Matthews, Thomas L Webb, Sarah Greenwood, Rosie Sharp, Carla Roberts-Owen, Eric Saldanha, Tom McBeth and Nicola J Buckland in Waste Management & Research

sj-docx-3-wmr-10.1177_0734242X251328964 – Supplemental material for Development and evaluation of an intervention to increase the collection of compostable packaging from households for industrial composting Supplemental material, sj-docx-3-wmr-10.1177_0734242X251328964 for Development and evaluation of an intervention to increase the collection of compostable packaging from households for industrial composting by Meghann Matthews, Thomas L Webb, Sarah Greenwood, Rosie Sharp, Carla Roberts-Owen, Eric Saldanha, Tom McBeth and Nicola J Buckland in Waste Management & Research

sj-docx-4-wmr-10.1177_0734242X251328964 – Supplemental material for Development and evaluation of an intervention to increase the collection of compostable packaging from households for industrial composting Supplemental material, sj-docx-4-wmr-10.1177_0734242X251328964 for Development and evaluation of an intervention to increase the collection of compostable packaging from households for industrial composting by Meghann Matthews, Thomas L Webb, Sarah Greenwood, Rosie Sharp, Carla Roberts-Owen, Eric Saldanha, Tom McBeth and Nicola J Buckland in Waste Management & Research

sj-docx-5-wmr-10.1177_0734242X251328964 – Supplemental material for Development and evaluation of an intervention to increase the collection of compostable packaging from households for industrial composting Supplemental material, sj-docx-5-wmr-10.1177_0734242X251328964 for Development and evaluation of an intervention to increase the collection of compostable packaging from households for industrial composting by Meghann Matthews, Thomas L Webb, Sarah Greenwood, Rosie Sharp, Carla Roberts-Owen, Eric Saldanha, Tom McBeth and Nicola J Buckland in Waste Management & Research

sj-docx-6-wmr-10.1177_0734242X251328964 – Supplemental material for Development and evaluation of an intervention to increase the collection of compostable packaging from households for industrial composting Supplemental material, sj-docx-6-wmr-10.1177_0734242X251328964 for Development and evaluation of an intervention to increase the collection of compostable packaging from households for industrial composting by Meghann Matthews, Thomas L Webb, Sarah Greenwood, Rosie Sharp, Carla Roberts-Owen, Eric Saldanha, Tom McBeth and Nicola J Buckland in Waste Management & Research

## Notes

1. We recognise compostable packaging that can be composted at home is also available, but the focus of the current research is the appropriate disposal of compostable packaging to be collected by the local authority to be industrially composted. As such, findings and recommendations provided here refer to industrial composting only and could be considered to be a limitation of the current work.

2. It has been proposed that by March 2026, all households in the United Kingdom will have a food waste collection – either as a standalone collection or mixed with garden waste if appropriate – as part of the Simpler Recycling Policy (DEFRA, 2024). The local authority involved in the study already offered a co-mingled food and garden waste collection.

3. For context, the food and garden waste bins were one of four outdoor bins households had access to. Alongside the food and garden waste bin, households had a black general waste bin for non-recyclable waste; a blue bag for recycling paper and cardboard and a white bag for recycling glass bottles and jars, metal food/drink cans, metal foil and plastic bottles, pots, tubs and trays (Medway Council, 2024).

4. The Index of Multiple Deprivation ranks small geographical areas in England, Wales and Scotland. Deciles are reported and range from ‘1 = most deprived’ to ‘10 = least deprived’.

5. Note that assessing the six COM-B components deviates from the pre-registered plan to assess three factors only. The results from the pre-registered plan are in Supplemental Material; however, we decided to present results on the six COM-B factors to provide greater detail on each of the main COM-B factors.

6. Based on the average weight of the compostable packaging items provided to households (2.5 g), however, there was variability in the weight of the packaging items provided and how they were disposed of (e.g. teabags weigh more being disposed of wet vs dry).
