# Supplementary material for: Development and evaluation of an intervention to increase the collection of compostable packaging from households for industrial composting
Source: Waste Manag Res. 2025 Apr 21;43(10):1636–49. doi: 10.1177/0734242X251328964 (PMC12476470; doi:10.1177/0734242X251328964)
Supplement: sj-docx-1-wmr-10.1177_0734242X251328964 – Supplemental material for Development and evaluation of an intervention to increase the collection of compostable packaging from households for industrial composting [file sj-docx-1-wmr-10.1177_0734242X251328964.docx]

# **Supplementary Materials**

## **Supplementary Material 1: Focus Groups: Methods, Results and Discussion**

### ***Design and procedure***

A qualitative phenomenological approach was used to address the main research question: what are the barriers and enablers to residents disposing of compostable packaging via their food and garden waste bin? To address this question, in five focus groups we explored residents’ attitudes towards compostable packaging and their perceived capability (e.g. awareness of compostable packaging), opportunity (e.g. access to food and garden waste bins) and motivation (e.g. benefits to composting packaging) to dispose of compostable packaging in the food and garden waste bin.

The minimum sample size was met as determined by recommendations for meeting data saturation (Hennink & Kaiser, 2022). Furthermore, no new information or themes were identified in the fifth focus group, indicating that data saturation had been met. Participants were recruited via purposive sampling using regional targeted social media advertisements^^[[1]](#footnote-0)^^. To participate in the focus groups, participants had to be at least 18 years old and live in Medway (Kent, UK) within an area where the food and garden waste bins were provided by the local authority. Specifically, based on Area Classification for Output Areas (Office for National Statistics, 2015), we targeted residents from suburbanite and hard-pressed living areas as these represent 46% of the areas in the local authority within which the intervention was to be conducted. Four focus groups were held in-person at a community centre in Rochester, Kent, and one was held online. A discussion guide was used which was structured to assess perceived capability, opportunity and motivation (see <https://osf.io/ztvku>).

At the start of the focus groups, a range of food and beverage items in compostable and non-compostable packaging were displayed on the table (e.g. supermarket carrier bags, tea bags, coffee cups, vegetable and confectionary packaging) and participants were asked to indicate which bin they would dispose of the packaging after use. Images of bins provided by Medway Council’s waste collection service (Medway Council, 2024) were shown, including: blue bins/bags used for recycling paper and card; white bins/bags used for recycling glass bottles and jars, metal food/drink cans, plastic bottles, tubs and trays; a black bin used for non-recyclable general waste and; a brown bin used for food and garden waste, with the last of these being the target bin for compostable packaging. A voting system was used whereby participants were asked to raise their hand to indicate which bin they would use to dispose of each item (in the online focus group, a digital polling tool was used). This task served two main purposes. First, it was a warm up exercise to help create an open and safe space to encourage participant discussions (Bates et al., 2017). Second, the items allowed us to demonstrate to participants examples of compostable packaging, to ensure that participants knew what the researchers meant by ‘compostable packaging’ and support the discussions on compostable packaging. After the exercise, compostable items were grouped together and participants were informed that the items were compostable. The focus groups lasted around 1 hour and were led by a researcher from the University of Sheffield (NJB) and attended by two researchers from Hubbub (a creative environmental charity; RS, ES). Upon completion, participants received a £20 shopping voucher for their time. The discussions were audio recorded (using a TASCAM DR-40 X) and transcribed verbatim by a third-party transcription service.

### ***Researcher characteristics and reflexivity***

The researcher who led the focus group discussion and conducted the thematic analysis is a White British female citizen who is highly educated and has conducted research related to behaviour change and sustainable waste behaviours. NJB is originally from Kent, the same council whereby the focus groups were conducted (different town). The researcher recognises that such positionality informs her understanding of the topic explored, the interaction with participants, and potentially the analysis and reporting of results.

### ***Results***

Thematic analysis following recommended procedures (Braun & Clarke, 2006; 2023) was conducted in NVivo by one researcher (NJB, inductive approach) and peer checked by another researcher (MM) who reviewed codes, themes and extracts. The identified themes were mapped onto the COM-B components (deductive approach) by two researchers (MM, NJB) through group discussion. Main themes generated by the researchers are shown in Table S1 with mapping to relevant COM-B factors and example quotes. Main themes were: identifying compostable packaging is challenging; labels for compostable packaging; guidance and resources from the council; a need for increased awareness and/or education about compostable packaging; storage space and/or access to bins is challenging; influence from others; Scepticism about the waste process; motivation to compost packaging; larger scale changes are needed. Each theme will now be discussed.

Table S1. Thematic codebook generated from focus groups.

| **Theme (COM-B model component)** | **Sub-theme** | **Barrier/**  **Enabler** | **Example quote(s)^a^** |
| --- | --- | --- | --- |
| **Identifying compostable packaging is challenging**  (COM-B: Psychological capability) | Difficulty identifying compostable packaging | Barrier | Participant 22: “*I mean, I would never be able to look at this and think this is compostable.”*  Participant 32: “*Yes, just looking at this one, it's not immediately clear to me that it is compostable. In fact, even now I'm not quite sure.”*  Participant 21: *“It seems better for the environment overall long-term, if it's compostable. But then packaging-wise, yes, hardly any of us I think would be able to identify it.* |
|  | It looks and feels like normal plastic | Barrier | Participant 6: *“I was really shocked when I saw compostable for that one because I thought this was like an ordinary one. I mean, I've read and I've heard about them but I didn't think it was going to be so, like, plasticky, you know?”*  Participant 3: *“It feels plastic, it clicks like plastic”*  Participant 5: *“I think there's part of me, I just still-, I don't believe it. I just, I don't…. Because I think that's with, you know, you had it drummed into you for so long that-, and obviously, the rubbish on the beach, this crisp packet is, you know, older than me. You know, it's 50 years old, so then how do you then get something that looks and feels exactly the same but different.”*  Participant 21: *“But I was a bit surprised, because I would never naturally think that plastic is going to be compostable. So I wouldn't even bother looking to be honest, I would have just dumped it with the plastic”*  Participant 44: *“…because I definitely would put that one in the white plastic* (recycling bag for plastic bottles, pots, tubs and trays) *without really thinking, because it's rigid. I'd put it straight in the white plastic one without thinking about it. I don't think I would normally look, but now I'm aware I should look at it more carefully.”*  Participant 31: *“Otherwise, something like this I wouldn't have is compostable. I'd just think it's plastic and straight into the white* (recycling bag for plastic bottles, pots, tubs and trays)*.”*  Participant 42: *“Yes, I think at a glance, they'd* (bin collectors) *look at that and think, 'That's plastic, and I'm not putting that in the compost waste.”* |
| **Labels for compostable packaging**  (COM-B: Physical opportunity) | Unclear labels on compostable packaging except for a few clear examples | Barrier | Participant 3: *“Most of them aren't marked very clearly.”*  Participant 1: *“They're all marked differently.”*  Participant 5: *“And if it says it, like, there and it's in your face. Whereas on that little plastic thing for the cookie, it is compostable but you wouldn't even look to look at that tiny writing.”*  Participant 32: *“I think the labelling could be quite easily missed. Then what it's actually telling us is, 'Made from plants. For cold food only.' That is pretty meaningless to me in terms of what I could do with it, you know, in terms of recycling or whatever.”*  Participant 5: *“The writing's underneath the food that you're eating, as well; P*articipant 3: *“Which, you're probably not going to see it.”*  Participant 3: *“You're trying to cook dinner, the kids are screaming and are you going to sit there on  every single packet? You know, that one stands out clear. Not many of the others are clear where they should go.”*  Participant 21:*“Apart from the Co-op bag actually, that was very very clear it's compostable.”*  Participant 24: *But  it's* (Co-op carrier bag) *like, written in big and it actually says what you can do with it and how you can use it, it's just very, very clear. And probably the fact, there's something about it being green, just now thinking about the conversation but I think the wording there is just clear, 'Don't put it in plastic,' and, 'Use it for compost.' It's really clear.*  Participant 32: *“But something like this one* (Rhythm 108 cookie) *where it is quite clear, 'Compostable 100%.' You know, that means something to me and I think I would know what to do with it.”*  Participant 32: *“This one, when we first looked at it, did actually jump out at me. And again, I know you said about the feel of something, I mean that was my first thought even before I actually looked at the labelling. It's something that kind of feels like it could be recycled.”* |
|  | Check packaging for labels and disposal instructions | Enabler | Moderator: *“Is checking instructions or symbols on packaging, to work out which bin it goes in, something that you do automatically?”;* Participant*:“No.”*  Participant 1: *Yes, I check things automatically. Some of the plastic packaging, like on the outside of the tea bags there, that can be a bit not recyclable yet. I think I read it quite carefully.*  Participant 24: *So yes, we look at it quite closely in our house and, you know, it's something that our little group encourage with other villages, and there has been a response.*  *Participant 53: Yes, I agree with that. And especially if you're buying something new that you've not bought before, you tend to look at the packaging to try and decipher as to what do with it.* |
|  | Clear and uniform labels that use a colour code |  | Participant 31: *“The symbols should be a lot easier, right? We should have some international system of a symbol and coding system or something because across these compostable ones, I cannot tell. Yes, so this has compostable 100% wrapper, this has compostable packaging so they're all quite different, aren't they?”*  Participant 44: *“I can think of a universal symbol. Quite big.”*  Participant 23: *“And at least have the symbol or colour on the front of the packaging, so it's less time to look at the back.”*  Participant 54: *“Maybe the words compostable packaging actually written somewhere on it clearly that you can actually see.” P*articipant 55: *“Yes, and specify that it can go in the food waste.”*  Participant 42: *“Well, I think it's up to the manufacturers actually to label their packaging better. There are thousands of products out there…. It's really up to the manufacturers and suppliers to label the packaging very clearly and distinctly and they all need to be synchronised in doing that. They all need to agree in a code of conduct marking their packaging because everyone's got their own ideas. If you had a symbol that was universal, we would all know where we are.”*  Participant 3:*“A traffic light system, 4 colours. Like you do on the (inaudible) or whatever. Like, a traffic light system…. A big black dot on it means it goes in the black bag. You don't have to read it then.”*  Participant 2:*“If there was a brown dot on it.”*  Participant 41: *“No, so a coloured symbol on there would make life a lot easier, wouldn't it?”*  Participant 53: *“If they gave us stickers, big stickers, we could stick them on the brown bin*  (food and garden waste bin) *so that every time you can say, 'This is the symbol for whatever compostable packaging is,' so that you don't have to then look online, you've got it somewhere big in the place where you're putting it”* |
| **Guidance and resources from the council**  (COM-B: Physical opportunity) | N/A | Enabler | Participant 24: *“But Medway do-, I mean, it's on their website, there's a whole list of things, if people have got nothing else to do with their lives. But they (the council) also distribute a little wheel where you can-, but obviously it's that size, and there's a limited number of items that are itemised around the edge. And you can see whether they need to go to household waste, blue bin, whatever, or if there are several possibilities. So there's some guidance.”*  Participant 31: *“I think so. Quite aware with the Medway Council. I think weekly or monthly newsletters, or they've promoted.”*  Participant 21:“*I think the* (waste collection) *bags are very good in helping identify what goes in each one, so I think that's very helpful.”*  Participant 43: *“For me personally, I only-, what is on the actually signage on the plastic bins is what I go by, and so I don't think everything could be compostable, I just chuck it in to whatever's on that packaging.”*  Participant 22: *“You can go over there and take one, nobody asks any questions. It's not a bad thing, but there isn't any additional support provided when you're taking the bags, or guidance”*  Participant 41: *“They're* (the council) *quite good at the moment about putting stuff out on Facebook. I saw a thing literally just this week that you can now recycle trigger sprays if you read it on the bottle which previously you couldn't. They are putting that sort of stuff out on Facebook. Then I guess I do look for it and, of course, things you look for come to you on Facebook, don't they?”*  Participant 52: *“I find my own, use my own guidance.”*  Participant 32:*“I've looked online and I have to say, Google's our friend, and quite often it's not Medway Council, it could be any… you sometimes find a local council that has really good guidance on recycling, really detailed guidance. Of course, if you Google something really specific it can take you anywhere, kind of thing… I tend not to think too much about what my local council is saying, but what I can find more broadly, more generally. I think that was actually what led me to discover, just Googling it, that there was actually a way of recycling coffee pods. Whereas, you know, I'm not sure that there's anything I would've found from the local council that would've told me that. So, it's a bit random to my mind.”*  Moderator: “*Okay and how about for actually disposing compostable packaging, have you received any guidance or information about which bins to use?*  Participant 55: “*I don't remember seeing anything.*” |
| **A need for increased awareness and/or education about compostable packaging**  (COM-B: Psychological capability) | N/A | Enabler | Participant 43:*“I think more information, more knowledge and posters from the council. They should be collaborating or something should be done to spread more awareness on where to put it. We as consumers, we'll just buy it. After today, everything here I would have thought I would have put them segregated in different bins, but when I realised that these are compostable, it's kind of changed my views on it. More awareness I think and councils should be educating their group of people in Medway. I know they've got loads of people, but just to give us more education and highlight these issues.”*  Participant 5:*“We could do with the codes being explained. Perhaps on a flyer, if they do a flyer at some stage to what you put in what bin. Under the brown bin* (food and garden waste bin)*, perhaps the list the codes on things.”*  Participant 22:*“I think it would be cool if there was a way to, I'm just thinking out loud here, if you could just scan something and it just goes, 'That's compostable.' I mean, the only thing is I'd rather just something reads that and goes, 'I recognise,' let's say, I don't know, like, a Nescafé box, 'Put that in this,' rather than me looking through different bits of packaging in different places to look at where the symbols are. That would be quite cool and I'd probably use that more. Don't know if it exists but that would be good.” P*articipant 24: “*Yes, I mean, that is doable. That's a brilliant idea.”*  Participant 43*:“I think more information, more knowledge and posters from the council. They should be collaborating or something should be done to spread more awareness on where to put it. We as consumers, we'll just buy it. After today, everything here I would have thought I would have put them segregated in different bins, but when I realised that these are compostable, it's kind of changed my views on it. More awareness I think and councils should be educating their group of people in Medway. I know they've got loads of people, but just to give us more education and highlight these issues.”*  Participant 23:*“And how much of this is taught in school? Just out of interest. I mean, I think that's the starting point, isn't it? Because kids today are going to be the future of tomorrow. We can do our bit but I think it's probably important to maybe lobby this for schools to include in syllabus. Because if children start at home then maybe parents might catch on and vice versa but you'd, kind of, reach out to all demographics.”*  Participant 24:*“Yes. And its a tricky thing, isn't it? I mean, children watch television and, you know, they know. And a lot of children are rally quite bright and they know this is their future and are concerned about it. I mean, they can be a way to do it the other way round. They can, sort of, go, 'Oh, let's recycle,' so they can be a force for change.”* |
| **Storage space and/or access to bins is challenging**  (COM-B: Physical opportunity) | Efficient storage space needed for compostable packaging | Barrier | Participant 1:*“But it's storage as well, isn't it? If you've got a lot of stuff you're collecting in week after week from the whole of (redaction) and it's all compostable, what are you going to do with it all while it's gradually breaking down? How are you going to store it all?”*  Participant 42:*“One thing that is a problem that which maybe councils, or whatever, should take consideration to, is people haven't always got the storage space in their homes to store up all this packaging. And that is a big problem for some people. I do actually have a decent cellar space which is, I can stick it all in a plastic sack down there, but, you know, if you've got a big family and you're a bit short on space, where do you store it? And that, I think, is an issue that hasn't really been dealt with properly.”*  Participant 31: *“So it's all in the kitchen, so in terms of compostable if it can somehow fit in with that then that would be easier. So I guess that's what originally happened because we used to put paper in that one and plastic in that one, but why do I have 2 bags when I can use the clear for both of them? So if that's the same with the food then yes that would be great.” P*articipant 32: *“Yes I agree, anything that, sort of, consolidates it's less room for error hopefully and just the space issue as well, it would be good.”*  Participant 21: *“It does mean a bit of effort. We've got a caddy but obviously then the caddy fills up so, where do you put that? Because that brown bin is right at the front. I've got a, sort of, intermediary bin, haven't I? That I do weeding and put incidental garden rubbish in and we usually” P*articipant 24: *“So he mixes it up”;* Returns to previous participant: *“When there's some of that, I'll put it in there for perhaps a day or so, and then it goes into the brown bin, so.”* |
|  | The food and garden waste bin is inconvenient for regular access |  | Participant 2: *“But it's a huge bin* (food and garden waste bin)*, so. It gets heavy, too heavy to move. I mean, I* could *leave mine 2, 3 weeks at a push. But this time of year, when you're doing more pruning…”*  Participant 32: *“And the brown bin is in our garden, which we then have to take all the way round the house, and you don't always want or need to put that out every week for collection. So it's not an ideal system. And also, I mean, you can't actually store that. It's a very small caddy isn't it?”*  Participant 32: *“Again just my personal view, I think something slightly not as unwieldy as the brown bin. And again I can't remember where but I think I've heard about other councils not so far away that have slightly different alternatives to the brown bin. If you've got 1, we've got 2, that's full of garden waste and grass they are actually quite heavy to move. And we have to go quite a circuitous route to get to the front and I don't know how old people would. You know, they might be able to put stuff in it but then they've got to actually physically so they probably-, it's not ideal.”*  *Participant 31: “my wife doesn't like the look of the brown bin. We have a 2 space driveway in front of the house so we put the bin somewhere where you can't really see it in front of the car, then we pick it up and wheel it out every morning, and as soon as we get home or when I get home I'll put it back. And then in the kitchen we have a bin for the plastics and paper and another bin for general waste and a food bin. So it's all in the kitchen, so in terms of compostable if it can somehow fit in with that then that would be easier. So I guess that's what originally happened because we used to put paper in that one and plastic in that one, but why do I have 2 bags when I can use the clear for both of them? So if that's the same with the food then yes that would be great.”*  Participant 21:*“But I think also the layout of the bins is quite interesting, because if I think back in London we only had 1 bin because of the lack of space, so everything went in the black bin.* Participant 21: “*Here we've kind of done 2 bins, so the majority of our paper and plastic currently goes in the recyclable bags that take everything, and then we stack up cardboard on the side and then put it in the blue one. But if the bins are a little bit further out, then you very naturally just-, so our black bin's close to the kitchen, we throw it in there. And then if we're really conscious about it we'll stack up a load of things and then put it in recycling separately. But we don't necessarily separate at least in 3, we don't even have the fourth one.”*  Participant 22: *“Again, I think a part of it is convenience, if the black bin's there you're going to-, like, your potato scrapping, if you just cut potatoes and then you go, 'Black bin, or brown bin outside. What am I going to do?' Or even the caddy, that's probably a little bit more convenient but it's just that whole thing of, where do you quickly throw something whilst your cooking?”* |
|  | Frequency put food and garden waste bin out |  | Participant 43: *“Yes, I mean, we use it every week because we've got a big family, so, every week is used. But it's never full to the brim.”*  Participant 23: *“Yes, it depends on the amount of cuttings etc from the garden, so it can vary from times of year. Obviously there are busy times when you're cutting stuff back, when you're cutting the grass. That would determine it rather than the actual waste that goes in it.”*  Participant 32: *“…how often it goes out is primarily to do with how much garden waste there is, which is what mainly builds it up. And I have to say, in terms of the food waste, that does maybe too often go in there. I mean, first of all we really do try to minimise food waste, which we're fairly good at. But well, it really just tends to go in the black bag, just discarded. The brown bin, it is quite unwieldy as I said. So it might even be like once a month or something.”* |
|  | No brown bin |  | Participant 22: “*I mean, I've read quite a bit on Facebook posts around people complaining about not having brown bins in flats, but we have a house and I'm pretty sure when we moved in January I requested all 3 of these and then ended up just coming here to pick them up, but they just don't get delivered. And don't they do 2 sizes? They do a big wheel-y one and a smaller one. But we just didn't get anything in there at all.”*  Moderator: *“Now that the council might start asking households to change and put compostable packaging in this brown bin, what do you think would make it easier for people to be able to follow that change?”;*  Participant 22: “*To have a brown bin.”;* Participant 23: *“Step number 1, yes.”* Participant 24: *“That would help.”* |
| **Influence from others Social others**  (COM-B: Social opportunity) | Family members support or disagree with general waste sorting | Barrier/enabler | Participant 43:*“I wasn't much before, but I did think about it more recently when I was living on my own and thinking of where to put what. Asking family as well because I was just totally unaware, so yeah. But still, I don't know if it was the right thing what I did at the time.”*  Participant 53: *“Yes, myself and my partner, I would copy what he does or vice versa. I'm like, yes definitely so.”*  Participant 31: *“Yes, I think, again, as we've started living together really recently, we have been conscious of where to put things and agree on that. So if I know that something is better placed somewhere else, and likewise she'll inform me.”*  Participant 4*“I normally tell my son off, because he always puts it in the wrong bin.”*  Participant 44: *“I go by, like you said, what's on the bags, but I have arguments with my older son who puts these in different places and I said, 'Look, that's recycling,' and he says, 'No it's not.' And he makes me read, so I've learnt more, I think, from having him around, obviously not quite enough.”*  Participant 21: *“I don't think people would say anything, or at least not our household. So I think if I was just to put, say, cardboard in the black bin, yes, I don't think anyone would say anything, or wouldn't even take it out.”*  Participant 6: *“They wouldn't do that. Because we are very united there, so we do what we're supposed to do.”* |
|  | Neighbours sorting waste |  | Participant 5: *“I mean, I go swimming in the early-, I say early mornings, so I see all the rubbish that's out when I go-, all the recycling's out. And it is very random as to what's in peoples’ bags of putting out their rubbish. Obviously some people are very precise about it, and they say it's-, and also the thing here, it's-, I don't know about other people.”*  Participant 56: *“I mean especially during Christmas, like having to sort of walk past a lot of areas that, because (redacted) we don't have an actual black wheelie bin, people just use bin bags and stuff for their own bins, and the amount of black bins I see like from one house. There's no recycling bags out. It kills me. I almost want to like, rip open their bin bags and start on their recycling because I'm just thinking, like, 'Surely you can.' There's so much to recycle in those bin bags. So it's affected me in a way but I think I'm quite confident of my recycling capabilities and abilities, so-,”*  Participant 31: *“I never see some of our neighbours' brown bins, so that could be a similar reason. So, we have had this conversation, me and my wife, our neighbours never put out their food bin. So, maybe they don't have as much food waste or they put it in the black bin. But they do put out the recyclable see-through bag, or the blue and white ones. So, I guess they're quite conscious of that area, so that's good. They have a family with kids as well, and so it does make a difference, the amount of waste they have compared to us.”*  Participant 42*: “I would put that on the other foot, I don't tell my neighbours. Although I know one side recycles but jumbles everything up and the other side don't recycle at all.”*  Participant 43 *“Yeah, same with me, I don't really see what my neighbour's doing, focusing on what my trash is.”* |
| Scepticism about the waste process  (COM-B: Reflective motivation) |  | Barrier | *Participant 53: “And again off topic, you sometimes see people on Facebook moaning about, 'Oh I've seen them, they mix it all in.' And I think, 'Well no I'm sure they don't. They don't mix it all in because they've probably got two halves on the bin bags.' Down our road the other week and I just happened to be working from home and ran out with some stuff, he had a big dumpy bag, you know, like you get your, I don't know your soil and stuff in, dragged it down the road and was just emptying all the bags into that one big bag. I think that's not good. It's not sending out the right message, even if you say, 'Oh yes, I know they've got the big sorty things at the end.' But actually if you're making an effort to sort your packaging, they should respect that and yes, there's plenty of room for a bin lorry in our road. There's no excuse for it. I can understand it in some of the narrower streets but there's not been an issue for the years we've lived here.”*  Participant 32: *“..I mean, like they're saying, I mean, not only test composting, but-, this is almost as big a concern I have in terms of, you know-, it's almost, sort of, fashionable to talk about greenwashing, but just how much of this really does get recycled in an environmentally friendly. I mean, if we're, you know, shipping off containers of plastic to the other side of the world, that's not very good either, but it might be better just to bury it or something. But yes, I mean, you have to hope and trust that it is composting and then re-used in terms of, sort of, prime materials, packaging, and so on.”*  Participant 22: *“I mean, to be honest, yes, we're recycling it, but whether that recycling's actually going to add value and somebody's really going to do what they're supposed to do down this recycling chain, I don't really know. And I don't 100% believe that's going to be done either, because you need the manpower, resources, and investment from the council to really be able to do that, and a lot of councils don't.”*  Participant 42:*“My question would be whether it would get emptied, because sometimes I've put stuff that I know to be compostable waste in my brown bin, and it hasn't been collected. And so I usually then, the following week, leave a note saying, 'Everything in this bin is compostable, please empty it.' But, yes, I mean I don't know, that if I had a little-, because I just have a little, brown-, so I don't know. It's that, sort of, size. If that was full of those, I'm not sure that the bin collectors would take it.”* Moderator: *“If they thought that it wasn't compostable?”* Participant 42: *“Yes, I think at a glance, they'd look at that and think, 'That's plastic, and I'm not putting that in the compost waste.”* |
| **Motivation to compost packaging**  (COM-B: Reflective and automatic motivation) | General environmental concerns | Enabler | Participant 44*:“It's really scary when you watch documentaries and they talk about the amount of plastic that's in the sea, and inside animals, and probably us as well.”*  Participant 44: *“I had a student lodger and she was appalled by the plastic wrapping that the fruit and vegetables come in in the supermarket. She said in Germany, it's all in paper bags and, 'We don't get that sort of plastic wrap.' I don't know what they do, but she didn't think you did.*  Participant 41: *“I've actively reduced my plastic packaging. So I go to (supermarket name redacted) more because they have more loose veg, and that sort of thing.”*  Participant 21:*“I think for me it's a matter of convenience still. It's that things just happen to come in a vessel, and if it was glass or metal or whatever else it could be better, but obviously there's an element of cost that the manufacturers have to look at. So it's a bit disturbing the amount of plastic there is there, but then what else could it be? There's so much convenience around the shampoo bottles, just picking one up. I know they do the refillables, but they also just come in bigger bits of plastic.”* |
|  | Motivated to compost packaging but mixed views on the benefits and environmental impact |  | Participant 52: *“I personally think it's* (compostable packaging) *better. I don't know how long each thing takes to compost, like to biodegrade but I think yes, like, at least we know it's not just going to sit in the environment and end up in some landfill and stuff.” P*articipant 51: *“I think it's better than using plastics definitely.”*  Moderator: *“And how motivated would you be if this change did come in? How motivated would you be to put compostable packaging in the brown bin?”* Participant 54: *“I would do it all the time.”*  Participant 21: *“It's not a gradual process, but it does require effort. Yes, it does require effort so I'm guessing not everyone would make that effort. But we make the effort, don't we?”*  Participant 3:*“How long does it take, because with these we're quite concerned that these are at least 20 years because we're still digging up, like, old chocolate bar wrappers because they have a space on the school grounds. So obviously if we start to dig down, it will-, the wind's blowing all the rubbish everywhere, so we're picking up really old labels of cans and all sorts. So it's definitely many years, and it still looks brand new.”*  Participant 41: *“There also the issue, isn't there, if compostable stuff goes to landfill then it generates methane, which isn't a good thing. Especially when it could rot down and, like you say, feed the soil.”; P*articipant 42: *“That's right, you don't want it in the landfill sites.”*  Participant 56: *“It is better than using plastic but at the end of the day it's still packaging.”*  Participant 53:*“Definitely. Absolutely, compost is the way forward. I really think it's just mad that people don't do it or don't do it in their brown bins or don't have a bin at home.”*  Participant 23: *“I'm not sure if that* (composting packaging) *will have a big enough impact really.”* |
|  | Confusion with Unclear on compostable packaging, the composting process and contamination |  | Participant 21: *“Well, just breaking down of all the materials. The organic matter would help to breakdown, I presume, the inorganic, so that it could be used as a much, I suppose, for people's gardens. It's about the heat generated, isn't it? That breaks it down. And then obviously you've got worms, etc in there that help the process.”*  Participant 51*: “I'm probably going to get tripped up with terminology here but I think it might be interchangeable with biodegradable, I think. I'm not sure. It sounds very similar.”*  Participant 53:*“Go in a big-, what do you call those composty tube things? Hot composty things. I can't remember what they're called. Industrial composting thing-, and then breaks down and then sieved at the end of it, hopefully. I mean, that actually made me wonder when you said, (redaction), about putting everything in the brown bin. If you put everything in a hot composting thing, the stuff that doesn't compost is still there and at the end of that period of time and if it could just be sieved out, can it not just be fished out and recycled then? Or is it too dirty to do that then?”*  Participant 56: “*I know it's not-, so yes I think maybe like, certain takeaway companies and stuff should just put that to-, again I don't know how it, the process, I don't know how long the process actually takes. If it's like a cardboard, a greasy cardboard, how long that takes to like, decompose into compost or whatever.”*  Participant 24:*“I think one of the things that would motivate-, because now, you know, this question of, 'So where does it actually go?' And I'm thinking, 'Oh gosh, I don't actually know. Why don't I know that? I should've asked that question.' But knowing that, it is hard work and so people will quite readily say, 'Oh it all goes to the same place. I've seen them and they put it all-,' and all of that, sort of, stuff. And, you know, I think the situation is better than what people think and so if people were shown where it goes-, in September, a lot of our waste goes to (redacted) I don't quite know why it goes to (redacted) but it goes to (redacted). And they have, they call it a 'Wonder Day', they have open day and they show people.”*  Moderator: *“What do you think happens when compostable packaging goes into the wrong bin?”;* Participant 21: *Well, it'll get thrown out, I suppose; P*articipant 24*: “Yes.”; P*articipant 21: “*Doesn't it get sorted on the, sort of, conveyor belt, and get thrown out? And then end up possibly being incinerated.”* Participant 24: *“And you can have 1 item in the wrong bag and it's then considered contaminated.”* Participant 21: “*What, really? Would that contaminate the whole bag?”* Participant 24: “*I think so.”;* Participant 22: *To be honest, I'd not thought as far as, after it leaves my house, what happens to it. But now I think about it, I wouldn't know. If I, just say I put this container in the white bag, quite honestly I've no idea what impact it could or would have. And I guess if I did know I would probably make more of a conscious-, like you said, like, that whole bag of plastic goes to waste, if I'm spending lots of time trying to separate my plastic and I put one bad piece of recyclable in there, then if that all goes to waste I'm like, 'What's the point?' And so I would make more of a conscious effort to make sure nothing compostable goes in the plastic.”* |
|  | We try our best but waste disposal errors happen. |  | Participant 24: “*It's all you can do. Guilt's not a very useful emotion, so yes if I get it wrong I don't feel guilty but just keep trying.”*  Participant 31: “*I think we all do it, so they won't be that fussed. You know, we try our best. Yes, I think we've both done it and we've both just turned a blind eye because it's just a silly, easy mistake.”*  Participant 42: “*Well I'd be a bit cross with myself but then I think, is it really my fault?”*  Participant 54: *“Yes, there would be a slight gasp moment where I'm like, 'Oh, God, I've done the wrong thing,' and I probably would have a sense of guilt.*  Participant 55: *“I think if I did it and I realised I did it before bin day I probably would put it into the right bin but I'd like to think that at some point it gets sorted anyway but I don't know.”* |
|  | Ways to increase motivation to compost packaging |  | Participant 2: *“So if they put something in (redaction), like, 'Over the past year we've recycled X amount and it's gone towards this, this, and this. And this is what happens to your garden waste, and these are the organisations or whatever that benefited and how they benefited.' Just so that you can see.”; P*articipant 6: *“That's an excellent idea.”*  Participant 22: *“Yes. So once it's been, I'm going to use the word compost which might be completely wrong, what happens to it then? Does the council sell it on to companies to sell as, like, things for gardens? Because I'm just thinking if somebody went, say like or someone went 'Hey, here's a bag of soil for your garden that you've made from composting,' that probably would get people to be like, 'Hey, I'm getting something back from this.” P*articipant 24: *“It makes it very real, as well.”;* Participant 22: “*It makes it very real if, like, someone at went, 'Here's something that, as (redacted), you've done and achieved,' that's really cool. Whereas I don't know, at the end of that, once it becomes compost, what happens to it?”*  Participant 22: *“I think no, I think it's actually lack of availability of information really to know what your role is and what impact you can have.”*  Participant 2: *“How many of those cups have been recycled compared to how many have just been chucked?”* |
| Larger scale changes are needed  (COM-B: Physical opportunity) | Different waste collection system across the UK | Barrier | Participant 24: *“One of the difficulties is, I mean, and this is problematic for the manufacturers, every area has a different system of recycling. So if they were nationwide policies that would help everybody. But to me, some sort of colour coding would sort it.”*  Participant 32: *“Plus as you say, people don't live in 1 council area all their lives and they move and then they move somewhere and find it's completely different to what they had before in terms of recycling. And then you just, sort of, learn the whole new process.”*  Participant 42: *“Some boroughs have got totally different policies and they provide you with 3 bins for different things. Not here, but they're doing it in certain parts of (redaction), for example. My partner lives in (redaction) and you have to slot it between this and that and that. Maybe 3 or 4 bins. In fact, I have seen pictures where in some boroughs they've got 4 bins all different colours for different things. We don't go that far here. Again, it's the council trying to anticipate what you're going to be using, whereas the manufacturers ought to make it clear what their products are. I think primarily, it's got to come from the manufacturers and suppliers to label up their packaging properly.”* |
|  | *More action is needed from industry, manufacturers and government* |  | Participant 42: *“Well, I think it's up to the manufacturers actually to label their packaging better. There are thousands of products out there. The council can't educate you on every one. It's really up to the manufacturers and suppliers to label the packaging very clearly and distinctly and they all need to be synchronised in doing that. They all need to agree in a code of conduct marking their packaging because everyone's got their own ideas. If you had a symbol that was universal, we would all know where we are.”*  Participant 2:*“And I suppose it's-, and again it would be another thing for shops or whatever, doing-, but I suppose in shops where you have the sections that are vegan or gluten free or whatever, the shops very clearly identify those-, you know, have those areas where that's the products that are. So maybe in, sort of, shops that-, I'm not saying you would group together all of the products that are in compostable packaging, but some way of having it, you know, on the shop shelf. You know, that it's more-, it's easier to pick out.”*  Participant 44:*“Or the government could just tell them I guess, couldn't they that they have to do it?” P*articipant 42: *“Well, the government's trying to do all sorts of stuff, isn't it? I mean (redaction), we're trying to get people to put their rubbish out on the correct day. We've been banging on with this for 5 months sticking leaflets through people's doors as volunteers because of the fly tipping and people putting things out on the wrong day. It doesn't even sort anything out. You've got a real cross section of people living in this area.”* |

*Note.*

^a^Text in italics font show quotes from participants. Text in non-italics font under ‘quotes’ is text inserted by the researcher to aid interpretation.

Where there are multiple Participant numbers within the same paragraph, this shows the interaction between participants.

Participant numbers range between 1 and 56 to protect the anonymity of the participants (consecutive participant identifiers were not used for the twenty-two participants).

#### ***Theme 1: Identifying compostable packaging is challenging***

This theme refers to participants highlighting the difficulty identifying compostable packaging. Two main sub-themes were generated by the researcher:

*Difficulty identifying compostable packaging*

Participants reported that there were no or limited distinctive markings that the packaging was compostable. This meant that the packaging could not be instantly recognised as compostable. One participant also raised issues around inconsistency with tea bags as some can and some cannot  be composted.

*It looks and feels like normal plastic*

Participants reported that the compostable packaging looked like conventional plastic with some expressing disbelief that it was not conventional plastics. For example, some reported how the packaging seemed too rigid to be compostable packaging. Others commented on how they would automatically associate the packaging with conventional plastic due to its similar attributes. Such disbelief and comments that compostable packaging looks and feels  like conventional plastics, aligns with previous qualitative feedback from participants (e.g. Buckland et al., 2024; Taufik et al., 2020). Due to compostable  packaging looking and feeling like conventional plastics, participants reported that they would assume the packaging was conventional plastic and therefore put it into the recycling bin for conventional plastic (without checking for waste disposal instructions). One participant also expressed concerns that the waste collectors would think the packaging was conventional plastics and therefore not take the packaging if it was in the food and garden waste bin. As such, difficulty identifying compostable packaging and distinguishing it from conventional plastics was a main barrier to disposing of compostable packaging in the food and garden waste bin. Please, n*ote*. This theme does not focus on labelling of compostable packaging, as this is represented in another theme.

#### ***Theme 2: Labels for compostable packaging***

*Unclear labels on compostable packaging except for a few clear examples*

This sub-theme referred to discussions of the compostable items that were displayed to participants during the focus groups, specifically related to the labels on packaging. Participants commonly reported that the packaging was either not labelled as compostable, or if it was labelled, the label was unclear. In instances where the label was unclear, this was due to the label either being too small, located in a place not easy to see or the labels differed between items. Some participants commented that the unclear labels made it a relatively demanding task to identify the packaging and correct disposal route. Given these demands, some participants discussed how it is unrealistic to expect people with limited resources, such as time or competing priorities, such as caring for children, to check each item to determine which bin to use.

However, despite most discussions focusing on unclear labels, there were some items or packaging attributes that participants highlighted for clearly communicating that the items were compostable. This included most commonly the grocery carrier bag (Co-op) whereby participants commented on it clearly stating it was compostable packaging and providing instructions on what to do with it. Another item that some participants commented on for being clear was a cookie (Rhythm 108) due to a label stating 100% compostable. However, not all participants agreed with some raising concern that this was stated in ‘*tiny writing*.’ As such, having a clear and consistent label was identified as a potential main enabler to support the disposal of compostable packaging into the food and waste bin.

*Check packaging for labels and disposal instructions*

Participants discussed whether or not they tended to check packaging items for labels and disposal instructions. Some participants reported not being in the habit of checking items. Others said they were in the habit of checking items but for familiar items they frequently use, they already know how to dispose of it and therefore automatically choose a bin for disposal based on this pre-existing knowledge (and not checking the packaging). Participants discussed how they were more likely to check packaging for unfamiliar or new items which they did not know how to dispose of. As such, having a label which clearly specifies which bin to use was identified as a potential main enabler to disposing of compostable packaging in the food and garden waste bin.

*The need for clear and uniform labels that use a colour code*

Given the unclear and inconsistent labels, multiple participants reported on the need for a uniform, consistent label to help identify compostable packaging. Some participants said having a label on the front-of-pack would be helpful, as well as using a colour coding scheme which aligns with bin prompts to identify compostable packaging (e.g. a coloured sticker for the bin which corresponds to the colour of the label).

#### ***Theme 3: Available resources or guidance***

Participants discussed a range of resources offered by the local council, including both online and physical resources. Online resources included the council’s website, a regular newsletter and social media. Physical resources included a waste sorting wheel which showed which bin to use for a range of different items. However, it was noted that compostable packaging was not on the waste sorting wheel resource. Participants also referred to the waste collection bags, which listed the items that should go in each waste stream, as helpful in deciding which bins to use. As such, clearly specifying that compostable packaging should go in the food and garden waste bin is one potential enabler, given the frequently reported use of the waste collection bags to inform waste disposal decisions. The local library was also referred to as a resource to collect caddy liners and waste collection bags. As such, participants tended to be aware of general waste sorting guidance from the council. However, some participants said they did not use the local council’s guidance and preferred to research themselves using other sources. Furthermore, when asked specifically about whether any guidance had been provided in relation to compostable packaging, participants reported that they were not aware of any.

#### ***Theme 4: A need for increased awareness and/or education about compostable packaging***

Participants offered some potential approaches to increasing the public’s awareness about compostable packaging as a way to support the identification and clear instructions on disposal routes for compostable packaging. The ideas offered aimed to simplify the sorting process. For example, one participant suggested having a digital app that allowed for packaging to be scanned and clear information on which bin to use to be provided. This idea was well received by other participants. Another participant suggested a flyer that could be attached to the food and garden waste bin which specified which items go into the bin.

Several other participants referred to the need for education for children on compostable packaging. Ideas around this were to educate the children of the future generation, as well children being the ones who then influence their parents’ waste behaviours.

#### ***Theme 5: Storage space and/or access to bins is challenging***

*Efficient storage space needed for compostable packaging*

A main barrier identified by participants was that households would need storage space to collect bulky compostable packaging and there were concerns that some households would not have sufficient space in their houses for this. Some participants referred to additional rooms or space they have to store compostable packaging (e.g. a cellar) but recognised other people might not have access to such space.  Some participants suggested potential solutions to the space barrier and raised that an efficient system which builds on existing waste systems within the house would be useful, rather than introducing new waste procedures or bins. One participant also commented on how they tend to use an intermediary bin to collect household waste before taking it out to the food and garden waste bin.

*The food and garden waste bin is inconvenient for regular access*

Multiple participants commented on the food and garden waste bin being inconvenient to access on a regular basis due to it being outside, it being heavy, needing to be moved around the outside house area and/or it not looking aesthetically appealing. Such challenges were discussed as potential barriers to accessing the food and garden waste bin to regularly dispose of compostable packaging. Similar to the previous sub-themes, some quotes in this theme referred to extending current household waste systems, rather than introducing new bins or approaches. Some participants also suggested that collecting compostable packaging in the kitchen, at the point where it is being used and disposed of, would be convenient. Use of the kitchen food caddy, which was already being used for food waste was suggested as a potential storage option. Similarly, some participants discussed how the convenience or ease of access to bins influenced the bins they use. For example, one participant referred to using the bin closest to them while cooking, rather than accessing the bin that should be used for food waste that requires more effort to access due to it being outside. As such, strategies that are convenient and do not involve regular access to bins that are inconvenient to access were identified as potential enablers to the appropriate disposal of compostable packaging.

*Frequency that the food and garden waste bin is put out for collection*

Most participants reported that they regularly put their food and garden waste bin out for collection. Some said they put the food and garden waste bin out either most or every week. Others commented on how they put the food and garden waste bin out more in the summer when there was more garden waste. However, some participants also reported not putting the food and garden waste bin out regularly due to not generating much food or garden waste. The regularity that the food and garden waste bin was taken out for kerbside collection, supported use of this bin to dispose of compostable packaging. 

*No brown bin*

Some participants reported that either themselves of other people did not have a brown bin. Having a brown bin was identified as a main, first step enabler to being able to put compostable packaging in the food and garden waste bin.

#### ***Theme 6: Influence from others***

*Family members support or disagree with general waste sorting*

Participants acknowledged and discussed the role that family members have on their general waste sorting behaviours. Some commented on family members being a source of support or information on how to sort waste, especially given how confusing waste sorting can be. Participants referred to the family generally and specific family members such as siblings and partners. Other participants reported on correcting other family members’ waste disposal behaviours, such correcting their adult children’s waste disposal behaviours. As such, from the discussions, it was clear that family members discuss waste disposal and either offer support or verbally attempt to correct the waste disposal behaviours of others.

When asked specifically what household members would say if compostable packaging was put in the wrong bin, there were mixed responses. Some participants said family members would not say anything. Others dismissed the idea about putting compostable packaging in the wrong bin as it was something that would not happen in their household.

*Neighbours sorting waste*

Beyond family members, some participants commented on noticing waste sorting behaviours of their neighbours or those in their neighbourhood area. Some participants expressed shock at the amount of general waste put out for kerbside collection that was not sorted for recycling. Others commented on comparisons between their own and their neighbours’ waste practices. Others said they did not notice much of what their neighbours did in relation to waste sorting and disposal and kept their own waste disposal practices private where they could. As such, some participants expressed awareness about what their neighbours’ weight disposal practices, especially when not sorted for recycling. However, others were less aware of their neighbours’ waste disposal behaviours. From this theme, it was clear that indicators about what social others in the neighbourhood or household do in relation to waste may be a potential enabler to support the disposal of compostable packaging into the food and garden waste bin.

#### ***Theme 7: Scepticism about waste process***

Multiple participants commented on beliefs that either they or other people had that waste collectors combined all waste – general and recycling waste into the same bin. Where participants believed such co-mingled waste was sorted later in the process, they said it was not useful for households to see this comingling of waste after all the efforts from households to sort waste. Some called for clearer communications that address this perceived co-mingling of collected waste to reduce it demotivating households to sort their waste.

There was also some scepticism expressed about whether councils and other stakeholders have sufficient resources to process all waste, including compostable packaging and produce valuable outputs. One participant said the system relies on having hope and trust that the waste management processors will process waste as expected for environmental benefits.

Participants also discussed waste collectors not collecting waste if they thought the waste had not been collected properly. This included references to the food and garden waste bin with some participants reporting uncollected waste if waste collectors thought it had materials in that should not be disposed of in the food and garden waste bin. Therefore, it is clear some households have some scepticism about whether waste that has been sorted remains segregated and whether it ends up being processed in environmentally beneficial ways.  There was also concern that collected waste may not be collected if the waste collectors think the waste has been inappropriately disposed of. As one participant reported, this may well be the case with compostable packaging given how similar it looks and feels to conventional plastics (see Theme 2). Such beliefs and concerns could be a barrier to disposing of compostable packaging in the food and garden waste bin.

#### ***Theme 8: Motivation to compost packaging***

*General environmental concerns*

Participants expressed concerns about the environmental impact of packaging as seen or heard about from multiple sources including television documentaries. Participants frequently referred to the concerning amounts of conventional plastics in circulation for everyday items (e.g. shampoo, fruit). Some participants reported strategies they use to reduce their use of plastic. However, some participants also reported on the convenience of conventional plastics and questioned the limited amount they can do about it as a consumer. Therefore, participants were aware of environmental impacts of conventional plastics and most expressed concerns over it.

*Motivated to compost packaging but mixed views on the benefits and environmental impact*

When asked if they would be motivated to compost packaging using the food and garden waste bin, participants said they would be motivated, but some acknowledged that not everyone would be. There were also mixed views about the potential impact of compostable packaging for reducing environmental impacts. Some participants perceived compostable packaging to be environmentally friendly and better than alternative conventional plastics, however, one commented that it still uses potentially unnecessary packaging. Others either expressed concerns about negative environmental impacts of compostable packaging or reported being unclear on the composting process. Concerns about potential negative impacts of compostable packaging included the time it takes for packaging to break down which could result in littering if the break down time is too long. Other participants commented on the risk of methane if compostable packaging is not disposed of appropriately and ends up at landfill. Some participants expressed beliefs that compostable packaging can make a beneficial impact on the environment, but others reported not knowing or said the impact would not be large enough.

*Confusion with compostable packaging, the composting process or contamination*

Some participants had a general idea about compostable packaging and the composting process. However, generally the processes around composting packaging were discussed with some uncertainty. Some participants questioned why they did not know and felt they should know. One participant also reported that it would be useful to share the composting process with the public, as without this understanding, excuses can be made for not appropriately sorting waste. Similarly, there was confusion around the issue of contamination and whether contaminated waste is sorted by waste processors at a later date. This led to discussion between participants questioning the impact that contamination has. Knowing about the consequences of contamination, and undermining efforts to sort packaging was highlighted as a potential enabler to appropriately sort and dispose of waste packaging.

*Feelings if waste is incorrectly disposed of: ‘we try our best’*

When asked about feelings if waste is incorrectly sorted, multiple participants referred to themselves and others trying their best. However, they acknowledged that mistakes are inevitable and there was not much use in experiencing negative emotions. Some participants said after a waste disposal error they would carry on with their planned activities as there is not much more they can do to address the error. Another participant also questioned whether it is really their fault if they have sorted waste incorrectly, implying the wider systems needs to improve to support appropriate waste disposal behaviours. However, there were also some participants who said they would feel guilty if they were aware that they had incorrectly disposed of waste, and where possible they would correct the error or rely on the belief that it will get corrected later in the waste management process. As such, while participants reported trying to appropriately dispose of waste, they also did not experience extreme negative emotions or regret if errors were made, and highlighted that others in the chain are also responsible for sorting the waste.

*Ways to increase motivation to compost packaging*

Participants suggested strategies to motivate people to compost packaging. One strategy was to show the end output or benefits of composting in relatable ways, such as showing people the compost generated from the composting process. Similarly, another participant commented on the potential value of highlighting the individual impact people’s actions have. Another idea was to provide feedback on composting rates.

#### ***Theme 9: Larger scale changes are needed***

*Different waste collection system across the UK*

A challenge identified by participants was different waste collection systems across the UK. Participants discussed how the number and colour of bins for different types of waste vary across different regions. Participants raised how this creates challenges when people move from one area to another and have to learn a new waste disposal system. Some participants also noted that the varied waste system across the UK also limits the potential for standardised, uniform labelling approaches which refer to specific bins. A such, some participants suggested that the waste system should be more uniform and consistent across the UK.

*More action is needed from industry, manufacturers and government*

Participants discussed the responsibilities of stakeholders beyond households who need to take action around labelling and promoting effective waste and composting systems. Several participants referred to the responsibilities that manufacturers or packaging producers have to clearly label products so it is clear to consumers which bin to use for disposal (see Theme 2). Some participants said that shops should more clearly signpost compostable packaging. Participants also referred to the Government for driving forward changes and improvements to the waste disposal system. As such, participants were aware that there are a number of stakeholders involved in the process of appropriately disposing of compostable packaging and it is not just the responsibility of households. As such, caution may be needed in any communications that aim to encourage households to put compostable packaging in the food and garden waste bin, to ensure households feel they are part of a wider system and the responsibility is not on them alone to ensure compostable packaging is industrially composted.

### ***Discussion***

The focus groups identified a number of barriers and enablers that can be targeted to encourage households to put compostable packaging in the food and garden waste bin. Some barriers align with previous research, such as confusion identifying compostable packaging as it looks and feels like conventional plastic (e.g. Buckland et al., 2024; Taufik, 2020), the need for clear labels that clearly specify the action or the appropriate waste stream to use (e.g. Allison et al., 2021a; Buckland et al., 2024; Closed Loop Partners’ Composting Consortium, & Biodegradable Products Institute, 2023; Purkiss et al., 2022; Taufik et al., 2020) and the use of distinctive, colour-coded systems (Allison et al., 2022c; Buckland et al., 2024), such as aligning the colours used on compostable packaging labels with the colours used in other communications such as bin signage. Similarly, the physical opportunity to access the food and garden waste bin was identified as a barrier to using this bin to dispose of compostable packaging, as it was positioned outside and could be heavy or inconvenient to access and move. Some participants also referred to having limited space in their households to store compostable packaging before disposing of it for collection. To address this barrier, some participants referred to having an efficient storage space, which builds on the existing physical home environment and is located at the point at which packaging is being disposed of (e.g., in the kitchen). This aligns with previous research showing that interventions which involve restructuring the physical environment tend to be effective at encouraging environmentally sustainable waste behaviours, such as recycling and composting food waste (Allison et al., 2022c; Bernstad, 2014; Sewak et al., 2021; Varotto & Spagnolli, 2017).

Another barrier discussed to the disposal of compostable packaging via the food and garden waste bin, was residents’ scepticism (or perceived scepticism other people had) about other key stakeholders in the waste system and the limited input that individuals have to support a sustainable waste system. This aligns with calls for a systems-wide approach to support sustainable waste systems and the important acknowledgement that multiple stakeholders’ are required, not individual behaviour alone (Iacovidou et al., 2021). Similarly, multiple participants acknowledged that while they try their best to engage in sustainable waste behaviours, due to challenges in the wider system (such as packaging not being clearly labelled), mistakes in waste sorting were inevitable. This perceived limited individual responsibility around waste sorting meant that some participants did not link waste sorting errors with negative emotions such as feeling guilty. Nonetheless, other discussions highlighted that participants were concerned about climate change and the excessive availability or perceived unnecessary use of conventional plastics used for items such as food packaging. Such consumer concerns over unnecessary use of conventional plastic packaging have been identified in previous research (e.g., Bauer, Dörnyei & Krauter, 2023). Due to such concerns, some discussions focused on the role that individuals were taking or had the motivation to take to reduce their impacts on the environment. This aligns with wider reports that consumers are shifting towards being more environmentally conscious and expressing preferences for sustainable packaging (Statista. 2022). As such, targeting key drivers, such as packaging attributes, labels, restructuring the physical environment (to make the desired behaviour the convenient option), providing indicators about social others’ waste behaviours and highlighting the relatable benefits (motivators) to composting packaging may be valuable targets for an intervention that aims to encourage consumers to dispose of compostable packaging in the food and garden waste bin that is collected for industrial composting.

*Strengths and limitations*

While the focus groups have a number of strengths, including recruitment of the target population for the planned intervention and holding the sessions in a community setting to promote uninhibited responses, a number of limitations need to be considered. First, it is possible that the focus groups attracted participants with a particular interest in environmentally sustainable behaviours. Indeed, some participants referred to specific environmental initiatives or actions they engage in, which may not reflect the wider target population. Despite this, there were a number of participants who expressed limited awareness of compostable packaging and environmental behaviours generally. Similarly, most participants expressed confusion with the composting process. Shifting to using the food and garden waste bin to compost packaging is also a new behaviour for the target population and therefore, the barriers and enablers identified offer valuable insights into the potential targets for intervention to encourage households to dispose of compostable packaging via their food and garden waste bin.

## **Supplementary Material 2: Intervention Development Meetings and Intervention Components**

In the first meeting, the barriers and enablers from the focus groups were discussed and ideas for interventions to address each barrier were generated using a shared online Mural board. The COM-B model (Michie et al., 2011) and the EAST (Easy, Attractive, Social, and Timely) framework (Behavioural Insights Team, 2012) were used to structure the discussions. Specifically, the COM-B model was used to identify whether the interventions would be targeting capability, opportunity and/or motivation, which in turn could allow the refinement of the intervention in line with guidance from the BCW (Michie et al., 2011, 2014). The principles from the Easy, Simple, Attractive and Timely Framework (EAST; Behavioural Insights Team, 2014) was used to generate initial intervention ideas. The EAST framework is an accessible framework that is suited for developing interventions within applied contexts.^^[[2]](#footnote-1)^^ EAST proposes that to encourage a targeted behaviour, the behaviour should be easy (e.g. have as the default, clear messages), attract attention (e.g. salient messages), be social (e.g. most people engage in or approve the behaviour) and timely (e.g. deliver interventions at the point decisions are made).

In the second meeting, initial intervention ideas were refined, the mode of delivery was discussed, along with the design of the evaluation intervention. Notably, at the point of the second meeting, only the COM-B model was used to inform intervention development. Ideas informed by the EAST Framework were transferred and mapped to the COM-B model. In the third meeting, the intervention components were selected for development and implementation. The selection of the intervention components was based on their potential effectiveness and the extent to which it was deemed feasible to implement the interventions within households and upscale if appropriate following the intervention.

Two intervention components were developed to address difficulty identifying compostable packaging (psychological capability), namely a front-of-pack label and a back-of-pack label. The front-of-pack label stated that elements of the packaging were compostable and directed households to check the back of the pack for disposal instructions (see Figure S1). The label was designed with a superhero theme that was used to support the coherency and engagement of the intervention components that were delivered to the households throughout the intervention. This theme was based on the barrier that compostable packaging can look and feel just like normal plastic and was used to frame as packaging in disguise, like a superhero.

The back-of-pack label had a logo and clear, action-based instructions on where to dispose of the packaging (i.e., ‘*Put in food waste bin’*, see Figure S2). Similar to Buckland et al. (2024), the label matched the colour of the bin that households were asked to put compostable packaging in. The design of the label was informed by input from On-Pack Recycling Labels (OPRL). The logo was a ‘C’ shape (for ‘compostable’) and designed to appear as if it was going into a bin. This design was selected from five options based on discussions with representatives of all project partners, reviewing results from previous compostable label studies (e.g., Allison et al., 2024; Buckland et al., 2024) and from online user testing with 200 participants, conducted by Hubbub. The compostable label was part of a dual label which featured the ‘Do Not Recycle’ label to ensure it was consistent with proposed labelling legislation for all packaging in the UK. The labels were modified to suit varying packaging needs (e.g. adapted to give correct disposal instructions for packaging with multiple materials; total of seven versions were created, see OSF). Both the front-of-pack and back-of-pack labels were applied as stickers (made of compostable materials) to all items provided in compostable packaging during the intervention period. Back-of-pack labels with accurate disposal information were also applied to items provided in non-compostable packaging, so that the presence of a back-of-pack label itself didn’t indicate that packaging was compostable.

To address barriers relating to the positioning of the food and garden waste bins that make the bins difficult to use (physical opportunity) and having insufficient storage space at home for compostable packaging (physical opportunity), households were provided with tips on how to position their food caddy^^[[3]](#footnote-2)^^ and their food and garden waste bin. The tips encouraged households to collect compostable packaging in their food caddy before taking it out to the food and garden waste bin. Households were also encouraged to position the bin in an accessible place in the kitchen: “keep your caddy in a handy, visible place, such as on your kitchen counter, so it’s easier to bin your food waste and any compostable packaging”. These tips were developed in line with guidance from Reijula and Hertwig (2020). Alongside being delivered in the leaflet (see Figure S3), these messages were also delivered electronically via text message during the intervention period.

Households were also provided with visual reminders to check packaging labels and use the food caddy and food and garden bin for compostable packaging. Specifically, households were provided with a paper band designed to look like a superhero eye mask to attach to the food caddy and a tag to attach to their food and garden waste bin (see Figure S4). The bin tag also served as a social cue to neighbours that households were participating in the trial and composting packaging.

Two intervention components were developed to address the barriers relating to the households’ lack of awareness of the composting process and the resulting outputs (reflective motivation); namely, an infographic and a bag of compost. The infographic outlined what happens when the compostable packaging is collected and taken to an industrial composting facility (e.g., how it breaks down), and for what purposes the resulting compost can be used (see Figure S5). This intervention component was delivered in the form of a leaflet which was attached to a bag of compost, which was delivered to the households. The bag of compost was intended to provide a visual representation of the resulting output from the process of composting, to make the outcomes salient and relatable.

## **Supplementary Material 3: Survey Measures**

The following questions were included in the pre-intervention survey:

*General recycling behaviours.* Participants were asked to rate the extent to which their household recycles (i) dry recyclables (i.e., plastic, glass, metal, cardboard, paper), and (ii) puts food waste (i.e., meat and fish, plate scrapings, fruit and vegetable peelings and cores, egg shells, tea bags, coffee grounds) in the food and garden waste bin. They were asked to rate these on a 6-point scale (1 = never, 6 = all items). For those who stated that they did put food waste in the food and garden waste bin, they were also asked to select which types of food waste they put in the food and garden waste bin (e.g., fresh produce with no packaging, fresh produce with packaging, any tea bags, tea bags only if they are labelled biodegradable/compostable).

*Attitudes.* Attitudes towards (i) recycling dry recyclables, and (ii) putting food waste in the food and garden waste bin were measured using the stem “For me, recycling dry recyclables / putting food waste in the food and garden waste bin is…” followed by 5-point response scales anchored by: foolish-wise, bad-good, harmful-beneficial, unenjoyable-enjoyable, unpleasant-pleasant, unfavourable-favourable, and negative-positive. This measure was derived from Ertz et al., (2017) who adapted the items from Sparks and Shepherd (1992). The items were internally reliable for attitudes towards recycling dry recyclables (α = .63) and attitudes towards putting food waste in the food and garden waste bin (α = .87). The items were combined into two separate indexes whereby higher scores indicate more positive attitudes.

*Other recycling questions.* Participants were asked a number of questions to provide context about their household recycling behaviours. These included being asked to select which of the following statement applies most closely to them: “I do not recycle at all”, “Recycling is a good thing but I don’t spend too much time worrying about it, I include the same things in the recycling every week”, or “Recycling is extremely important and I take the time and effort to ensure that I’m doing everything right”. Furthermore, they were asked who in the household is responsible for recycling (open text response), how old the person / people who are responsible for recycling are, and whether everyone in the household recycles to the same extent (yes, no, sometimes) and to briefly explain their answer. Households were also asked to rate on a 5-point scale how confident they are in selecting which bin to put the recycling in (1 = very unconfident, 5 = very confident).

*Pro-environmental identity and concern.* Pro-environmental identity was measured using 4-items from Whitmarsh and O'Neill's (2010) Green Identity Scale. Examples of items are “I think of myself as an environmentally friendly consumer” and “I would be embarrassed to be seen as having an environmentally friendly lifestyle”. The items were internally reliable (α = .64) and so were combined into a single index. Pro-environmental concern was measured using 4-items developed by Ellen (1994) such as “Compared to other things in my life, environmental problems are not that important to me”. The items were internally reliable (α = .70) and so were combined into a single index. Participants responded to all these items on a 5-point scale (1 = strongly disagree, 5 = strongly agree). Higher scores indicate greater pro-environmental identity and concern.

The following questions were included in the post-intervention survey:

*Food caddy use.* Participants were asked the frequency with which they put (i) food waste and (ii) compostable packaging in a food caddy, on a 6-point scale (1 = never, 6 = always).

*Questions about environmental restructuring intervention.* Participants were asked whether they had made any changes to (i) where they keep their food caddy or kitchen bins and/or (ii) where they keep their bins outside to make them easier to use and to describe these changes. They were also asked whether there is anything stopping them making changes to where they keep their food caddy or bins and if so, to describe these changes. Participants were also given the option of sharing some images of how they store their bins with the research team.

**Table S2.** *Survey items and internal reliability scores for COM-B components.*

|  | **Cronbach’s alpha** | | |
| --- | --- | --- | --- |
| **Survey items for each COM-B component** | **Pre-** | **Post-** | **Follow-Up** |
| **Psychological capability** | .69 | .74 | .76 |
| I am aware that some packaging is compostable  I don’t know what to do with compostable packaging after using it  I know what packaging I can and can’t put into the food and garden waste bin  I am aware that some packaging has multiple parts (e.g., a film and cardboard sleeve) and that not all of the packaging can be disposed of in the food and garden waste bin  I find it difficult to identify if packaging is compostable  I find deciding where to dispose of compostable packaging confusing |  |  |  |
| **Physical capability** | .57 | .55 | .81 |
| I find it difficult, physically, to put my food and garden waste bin out for collection  I find it difficult, physically, to sort compostable packaging to put in the food and garden waste bin |  |  |  |
| **Physical opportunity** | .71 | .77 | .78 |
| I have received clear information about what waste to put in each bin  Of all of my bins, it is clear which bin is for compostable packaging  The compostable packaging available is clearly labelled as ‘compostable packaging’  I can easily tell which bin compostable packaging goes in just by looking at the packaging  I have space in my kitchen/home to store compostable packaging before putting it in the food and garden waste bin  How I store my bins outside makes it easy for me to put compostable packaging in the food waste bin  The layout of my kitchen makes it easy for me to put compostable packaging in the food and garden waste bin |  |  |  |
| **Social opportunity** | .63 | .63 | .62 |
| There is a strong culture in my local area to put compostable packaging in the food and garden waste bin  Most of my neighbours/family and friends/the people I know put compostable packaging in the food and garden waste bin |  |  |  |
| **Reflective motivation** | .67 | .76 | .73 |
| Nothing useful is done with compostable packaging that is put in the food and garden waste bin  It does not matter if you put non-compostable packaging in the food and garden waste bin  It won’t make a difference whether I put compostable packaging in the food and garden waste bin or the general waste bin after using it  It is my responsibility to put compostable packaging in the food and garden waste bin after using it  Putting compostable packaging in the food and garden waste bin benefits the environment  I understand how compostable packaging is composted  I know what is produced by industrial composting |  |  |  |
| **Automatic motivation** | .68 | .71 | .73 |
| Putting compostable packaging in the food and garden waste bin is something that I do without thinking  I’m too busy to sort waste  When packaging has multiple parts, I separate out the different parts and dispose of the compostable part in the food and garden waste bin without thinking |  |  |  |
| **Capability** | .71 | .78 | .80 |
| **Opportunity** | .75 | .77 | .79 |
| **Motivation** | .77 | .84 | .81 |

**Table S3**. Participant characteristics (*n* = 86)

| Age | M = 56.08, SD = 15.18, Range = 23 – 85 years |
| --- | --- |
| Gender | Male = 14 (16%)  Female = 70 (81%)  Non-binary / third gender = 1 (1%)  Prefer not to say = 1 (1%) |
| Accommodation | Detached house = 2 (2%)  Semi-detached house = 54 (63%)  Terraced house = 30 (35%) |
| Relationship | Married = 51 (59%)  Civil partnership = 1 (1%)  Cohabiting = 16 (17%)  Single = 10 (11%)  Other = 7 (8%)  Prefer not to say = 1 (1%) |
| Number of people living in household | M = 3.04, SD = 1.36, Range = 1 - 7 |
| Households with children | 33 households had children  1 child = 12 (36%)  2 children = 12 (36%)  3 children = 8 (24%)  5 children = 1 (3%) |
| Ethnicity | Asian or Asian British = 1 (1%)  Black, African, Caribbean, or Black British = 3 (4%)  Mixed or multiple ethnic groups = 2 (2%)  White = 78 (91%)  Prefer not to say = 2 (2%) |
| Education | No formal qualifications = 11 (13%)  1-4 GCSEs or equivalent qualifications = 8 (9%)  5 GCSEs or equivalent qualifications = 15 (17%)  Apprenticeship = 4 (5%)  2 or more A-levels or equivalent qualifications = 19 (22%)  Bachelors degree or equivalent = 17 (20%)  Doctoral or higher education = 3 (4%)  Other qualifications including foreign qualifications = 3 (4%)  Prefer not to say = 6 (7%) |
| Employment | Full time = 24 (28%)  Part time = 19 (22%)  Unemployed = 2 (2%)  Student = 0 (0%)  Retired = 34 (40%)  Homemaker = 1 (1%)  Unable to work = 1 (1%)  Other = 3 (4%)  Prefer not to say = 2 (2%) |
| Annual household income (before tax) | Below 10k = 2 (2%)  10k – 24, 999 = 9 (11%)  25k – 34, 999 = 5 (6%)  35k – 44, 999 = 4 (5%)  45k – 54, 999 = 8 (9%)  55k = 64, 999 = 9 (11%)  65 – 74, 999 = 4 (5%)  75k or more = 7 (8%)  Prefer not to say = 38 (44%) |
| Pro-environmental identity | M = 4.14, SD = .61 |
| Pro-environmental concern | M = 4.03, SD = .63 |

*Note.* Pro-environmental identity and pro-environmental concern were measured on 5 point scales where higher scores indicate higher pro-environmental identity and concern.

##

## **Supplementary Material 4: Assumption Checks**

*MANOVA for COM-B subcomponents.* Preliminary assumption checking revealed that there were (i) there were linear relationships, as assessed by scatterplots, and (ii) there was no multicollinearity, as assessed by Variance Inflation Factors (defined as VIF < 5). Not all data were normally distributed (as assessed by Shapiro Wilk test), however, MANOVA is robust to violations of normality. A number of univariate and multivariate outliers were detected, as assessed by Z-scores (defined as |z| ≥ 3.29) and Mahalanobis distance (using the critical value of 28.87), respectively. In line with the pre-registration, these outliers were removed from the data and the resulting sample size was *N* = 74.

*ANOVA for self-reported disposal of compostable packaging.* Preliminary assumption checking revealed that there were no outliers, as assessed by Z-scores (defined as |z| ≥ 3.29) and while not all data were normally distributed, ANOVA is thought to be robust to these violations. Additionally, the assumption of sphericity was not met, as assessed by Mauchly's test of sphericity, χ2(2) = 22.05, *p* < .001, and thus was corrected using Greenhouse-Geisser.

*ANOVA for the online behavioural choice task.* Preliminary assumption checking revealed that there were no outliers, as assessed by Z-scores (defined as |z| ≥ 3.29) and while not all data were normally distributed, ANOVA is thought to be robust to these violations. Additionally, the assumption of sphericity was not met, as assessed by Mauchly's test of sphericity, χ2(2) = 35.02, *p* < .001, and thus was corrected using Greenhouse-Geisser.

*ANOVA for the online behavioural choice task - confidence in choices.* Preliminary assumption checking revealed that there were two outliers as assessed by Z-scores (defined as |z| ≥ 3.29) and while not all data were normally distributed, ANOVA is thought to be robust to these violations. In line with the pre-registration, outliers were removed (*N* = 84). Additionally, the assumption of sphericity was not met, as assessed by Mauchly's test of sphericity, χ2(2) = 11.22, *p* < .001, and thus was corrected using Greenhouse-Geisser.

**Perceived capability, opportunity, and motivation**

Preliminary assumption checking revealed that there were (i) no multivariate outliers, as assessed by Mahalanobis distance (using the critical value of 16.92) , (ii) there were linear relationships, as assessed by scatterplots, and (iii) there was no multicollinearity, as assessed by Variance Inflation Factors (defined as VIF < 5). Not all data were normally distributed (as assessed by Shapiro Wilk test), however, MANOVA is robust to violations of normality. In terms of univariate outliers, there was one outlier detected (as assessed by Z-scores, defined as |z| ≥ 3.29), and in line with the pre-registration were removed from the data. Table S4 shows the descriptive statistics for capability, opportunity and motivation to identify and dispose of compostable packaging over the course of the intervention. The MANOVA revealed that perceived capability, opportunity and motivation changed significantly over the course of the intervention, *F*(6, 79) = 6.25, *p* < .001; Wilks' Λ = .678; partial η^2^ = .322. Specifically, univariate ANOVAs and Bonferroni-corrected follow-up analyses (see Table S5) highlighted that capability, opportunity and motivation increased between pre-intervention and follow-up. Additionally, there were significant increases between pre-intervention and post-intervention for perceived capability and motivation (not opportunity), but there were significant changes for perceived opportunity between post-intervention and follow-up.

**Table S4.** *Means (and standard deviations) for capability, opportunity, and motivation for identifying and disposing of compostable packaging over the course of the intervention (n = 85)*

|  | Pre-intervention | Post-intervention | Follow-up | *p* | partial η^2^ |
| --- | --- | --- | --- | --- | --- |
| Capability | 3.88 (.63)^a^ | 4.01 (.66)^b^ | 4.14 (.67)^cb^ | <.001 | .095 |
| Opportunity | 3.39 (.71)^a^ | 3.49 (.66)^ab^ | 3.67 (.68)^c^ | <.001 | .108 |
| Motivation | 3.80 (.62)^a^ | 4.05 (.62)^b^ | 4.12 (.56)^cb^ | <.001 | .205 |

^abc^ = Within a row, means without a common superscript significantly differ (*p* < .05) based on Bonferroni-corrected follow-up analyses

**Table S5.** Mean difference, confidence intervals and p-value for Bonferroni-corrected follow-up analyses.

|  | Pre-Intervention - Post-Intervention | | | Pre-Intervention - Follow-Up | | | Post-Intervention - Follow-Up | | |
| --- | --- | --- | --- | --- | --- | --- | --- | --- | --- |
|  | Mean difference | 95% CI | *p* | Mean difference | 95% CI | *p* | Mean difference | 95% CI | *p* |
| Capability | .14 | [.009, .279] | .033 | .25 | [.091, .415] | <.001 | .11 | [-.035, .253] | .204 |
| Opportunity | .10 | [-.049, .250] | .312 | .28 | [.104, .455] | <.001 | .18 | [.047, .311] | .004 |
| Motivation | .25 | [.124, .375] | <.001 | .31 | [.177, .442] | <.001 | .06 | [-.045, .165] | .504 |

**Table S6.** Mean difference, confidence intervals and p-value for Bonferroni-corrected follow-up analyses.

|  | Pre-Intervention - Post-Intervention | | | Pre-Intervention - Follow-Up | | | Post-Intervention - Follow-Up | | |
| --- | --- | --- | --- | --- | --- | --- | --- | --- | --- |
|  | Mean difference | 95% CI | *p* | Mean difference | 95% CI | *p* | Mean difference | 95% CI | *p* |
| Psychological Capability | .21 | [.061, .354] | .003 | .32 | [.140, .490] | <.001 | .11 | [-.049, .265] | .285 |
| Physical Capability | .11 | [-.116, .332] | .722 | .01 | [-.205, .232] | 1.00 | .10 | [-.207, .396] | 1.00 |
| Physical Opportunity | .12 | [-.052, .295] | .271 | .10 | [.050, .340] | <.001 | .20 | [.050, .340] | .004 |
| Social Opportunity | -.02 | [-.261, .221] | 1.00 | .03 | [-.226, .293] | 1.00 | .05 | [-.118, .227] | 1.00 |
| Reflective Motivation | .30 | [.156, .439] | <.001 | .37 | [.215, .519] | <.001 | .07 | [-.048, .187] | .454 |
| Automatic Motivation | .17 | [-.034, .376] | .133 | .22 | [.008, .433] | .039 | .05 | [-.133, .232] | 1.00 |

**Table S7.** *Total number of households (out of 119) from which the food & garden waste and dry mixed recycling bins were collected at each week of the intervention.*

|  | **Week** | | | | | |  |
| --- | --- | --- | --- | --- | --- | --- | --- |
|  | 1 | 2 | 3 | 4 | 5 | 6 | **Average** |
| Food & Garden Waste (n) | 73* | 60 | 68 | 60 | 77 | 64 | 67 |
| Food & Garden Waste (*%)* | *61.34* | *50.42* | *57.14* | *50.42* | *64.71* | *53.78* | *56.30* |
| Dry Mixed Recycling (n) | 96 | 90 | 85 | 87 | 97 | 90 | 90.83 |
| Dry Mixed Recycling *%* | *80.67* | *75.63* | *71.43* | *73.11* | *81.51* | *75.63* | *76.33* |

*Note.*

*In week 1, waste was assessed from fewer households due to an error by the waste collection team which co-mingled the food and garden waste bin with other waste streams.

The dry mixed recycling waste was collected from blue and white bags intended for paper and cardboard (blue bag) and glass, plastic and metal).

**Table S8.** *Overview of data from waste audits*

|  | Week | | | | | |
| --- | --- | --- | --- | --- | --- | --- |
|  | 1 | 2 | 3 | 4 | 5 | 6 |
| Number of houses | 33* | 60 | 68 | 60 | 77 | 64 |
| Compostable (g) | 730 | 680 | 2730 | 410 | 1188 | 3516 |
| Organic waste (i.e., garden waste) (g) | 280000 | 460000 | 440000 | 400000 | 610000 | 380000 |
| Food | 11512 | 61204 | 88792 | 116491 | 103348 | 109140 |
| Food % | 4.11% | 13.31% | 20.18% | 29.12% | 16.94% | 28.72% |
| Contamination | | | | | | |
| % of contamination | 9% | 7% | 7% | 5% | 8% | 3% |
| Paper/Card | 13.36% | 35.12% | 32% | 67.18% | 81.41% | 41.53% |
| Textile | 7.03% | 0.70% | 0% | 0% | 0.89% | 11.59% |
| Wood | 72.64% | 30.65% | 0% | 8.48% | 6.89% | 0.09% |
| Glass | 1.27% | 0% | 6.03% | 9.39% | 1.68% | 16.92% |
| Other | 2% | 15.64% | 53.58% | 5.5% | 2.32% | 13.15% |
| Metal | 1.91% | 1.28% | 2.61% | 0.95% | 0.93% | 2.18% |
| Plastic | 2.80% | 16.60% | 5.80% | 8.50% | 5.87% | 14.53% |
| Proportion of waste by household | | | | | | |
| Number of houses | 33* | 60 | 68 | 60 | 77 | 64 |
| Compostable (g) | 22.12 | 11.33 | 40.75 | 6.83 | 15.43 | 54.94 |
| Organic waste (g) | 8484.85 | 766.67 | 6470.59 | 6666.66 | 7922.08 | 5937.50 |
| Food | 348.85 | 1020.07 | 1305.76 | 1941.62 | 1342.18 | 1705.31 |

* 73 households presented their food and garden waste for collection, however the data could only be analysed based on the waste for 33 households due to a collection error whereby food and garden waste was co-mingled with other waste streams for some households.

# **Supplementary Material 5: Last Observation Carried Forward - Results**

## **Survey Responses**

As outlined in the pre-registration, to handle missing data, the analyses were conducted with the last observation carried forward. For these analyses to be conducted, participants had to have completed at least the first survey, (*n* = 112). Of these participants, two participants failed attention checks and one participant provided suspicious data (i.e., provided the same response to every item). Therefore, the following analyses are based on data from 109 participants.

### ***Changes in perceived capability, opportunity, and motivation***

Table S9 shows the descriptive statistics for capability, opportunity and motivation to identify and dispose of compostable packaging over the course of the intervention. The MANOVA revealed that perceived capability, opportunity and motivation changed significantly over the course of the intervention, *F*(6, 95) = 6.14, *p* < .001; Wilks' Λ = .72; partial η^2^ = .279. Specifically, univariate ANOVAs and Bonferroni-corrected follow-up analyses (see Table S9) highlighted that capability, opportunity and motivation increased between pre-intervention and follow-up. Additionally, there were significant increases between pre-intervention and post-intervention for perceived capability and motivation (not opportunity), but there were significant changes for perceived opportunity between post-intervention and follow-up.

**Table S9.** *Means (and standard deviations) for capability, opportunity, and motivation for identifying and disposing of compostable packaging over the course of the intervention (n = 85)*

|  | Pre-intervention | Post-intervention | Follow-up | *p* | partial η^2^ |
| --- | --- | --- | --- | --- | --- |
| Capability | 3.79 (.66)^a^ | 3.92 (.71)^b^ | 4.05 (.72)^cb^ | <.001 | .079 |
| Opportunity | 3.37 (.69)^a^ | 3.45 (.70)^ab^ | 3.61 (.73)^c^ | <.001 | .077 |
| Motivation | 3.72 (.63)^a^ | 3.94 (.65)^b^ | 4.02 (.60)^cb^ | <.001 | .185 |

^abc^ = Within a row, means without a common superscript significantly differ (*p* < .05) based on Bonferroni-corrected follow-up analyses

**Table S10.** Mean difference, confidence intervals and p-value for Bonferroni-corrected follow-up analyses.

|  | Pre-Intervention - Post-Intervention | | | Pre-Intervention - Follow-Up | | | Post-Intervention - Follow-Up | | |
| --- | --- | --- | --- | --- | --- | --- | --- | --- | --- |
|  | Mean difference | 95% CI | *p* | Mean difference | 95% CI | *p* | Mean difference | 95% CI | *p* |
| Capability | .13 | [.016, .234] | .018 | .20 | [.066, .335] | .001 | .08 | [-.035, .187] | .303 |
| Opportunity | .08 | [-.043, .208] | .337 | .21 | [.062, .358] | .002 | .13 | [.027, .228] | .008 |
| Motivation | .20 | [.102, .294] | <.001 | .26 | [.151, .336] | <.001 | .06 | [-.028, .149] | .301 |

#### ***Exploratory analyses***

Table S11 shows the descriptive statistics for perceived psychological and physical capability, physical and social opportunity, and reflective and automatic motivation to identify and appropriately dispose of compostable packaging over the course of the intervention. A repeated measures MANOVA was conducted with the six COM-B components as dependent variables and time (pre-intervention, post-intervention, follow-up) as the within-subjects factor. The MANOVA revealed that the changes in the 6 COM-B components over the course of the intervention were statistically significant, *F*(12, 86) = 4.54, *p* < .001; Wilks' Λ = .612; partial η^2^ = .388. Specifically, univariate ANOVAs and Bonferroni-corrected follow-up analyses (see Table S12) highlighted that there were significant increases in perceived psychological capability, physical opportunity, reflective motivation, and automatic motivation between pre-intervention and follow-up. Additionally, there were significant increases between pre-intervention and post-intervention for psychological capability and reflective motivation, and between post-intervention and follow-up for physical opportunity.

**Table S11.** *Means (and standard deviations) for capability, opportunity, and motivation for identifying and disposing of compostable packaging at pre-, post-intervention and follow-up (n = 98)*

|  | Pre- intervention | Post- intervention | Follow-up | *p* | partial η^2^ |
| --- | --- | --- | --- | --- | --- |
| Psychological capability | 3.74 (.66)^a^ | 3.93 (.65)^b^ | 4.01 (.71)^cb^ | <.001 | .129 |
| Physical capability | 4.16 (.95) | 4.24 (.93) | 4.28 (1.00) | .341 | .011 |
| Physical opportunity | 3.53 (.71)^a^ | 3.62 (.78)^ab^ | 3.78 (.82)^c^ | <.001 | .087 |
| Social opportunity | 3.01 (.80) | 3.06 (.56) | 3.09 (.52) | .777 | .003 |
| Reflective motivation | 3.77 (.59)^a^ | 4.01 (.61)^b^ | 4.08 (.60)^cb^ | <.001 | .219 |
| Automatic motivation | 3.68 (.87)^a^ | 3.84 (.80)^ab^ | 3.89 (.80)^cb^ | .003 | .057 |

^abc^ = Within a row, means without a common superscript differ (*p* < .05) based on Bonferroni-corrected follow-up analyses

**Table S12.** Mean difference, confidence intervals and p-value for Bonferroni-corrected follow-up analyses.

|  | Pre-Intervention - Post-Intervention | | | Pre-Intervention - Follow-Up | | | Post-Intervention - Follow-Up | | |
| --- | --- | --- | --- | --- | --- | --- | --- | --- | --- |
|  | Mean difference | 95% CI | *p* | Mean difference | 95% CI | *p* | Mean difference | 95% CI | *p* |
| Psychological Capability | .19 | [.074, .314] | <.001 | .27 | [.131, .416] | <.001 | .08 | [-.041, .200] | .328 |
| Physical Capability | .08 | [-.101, .264] | .836 | .12 | [-.116, .351] | .672 | .04 | [-.141, .213] | 1.00 |
| Physical Opportunity | .09 | [-.054, .234] | .389 | .25 | [.085, .414] | .001 | .16 | [.042, .276] | .004 |
| Social Opportunity | .03 | [-.221, .170] | 1.00 | .06 | [-.167, .280] | 1.00 | .03 | [-.121, .182] | 1.00 |
| Reflective Motivation | .25 | [.137, .358] | <.001 | .32 | [.197, .442] | <.001 | .07 | [-.026, .169] | .234 |
| Automatic Motivation | .16 | [-.001, .321] | .052 | .21 | [.043, .358] | .009 | .05 | [-.090, .199] | 1.00 |

### ***Self-reported disposal of compostable packaging***

A one-way ANOVA revealed that the intervention had a significant effect on the amount of compostable packaging that households reported disposing of, *F*(2, 214) = 16.89, *p* < .001; partial η^2^ = .136. Bonferroni-corrected follow-up analyses showed that there was a significant increase in the amount of compostable packaging that households reported disposing of between the pre- (*M* = 3.88, *SD* = 1.84) and post-intervention phase (*M* = 4.46, *SD* = 1.49; mean difference = .58, 95% CI [.201, .966], *p* = .001) and between the pre-intervention and follow-up phase (*M* = 4.68, *SD* = 1.45; mean difference = .80, 95% CI [.413, 1.180], *p* < .001). There was no significant difference between the post-intervention and follow-up phase (mean difference = .21, 95% CI [-.039, .465], *p* = .127).

### ***Online behavioural choice task***

A one-way ANOVA revealed a significant effect of time on the number of trials that participants correctly selected the food and garden waste bin as a way of disposing of compostable packaging *F*(2, 216) = 55.06, *p* < .001; partial η^2^ = .338. Bonferroni-corrected follow-up analyses highlighted that there was a significant increase in the number of trials (out of 14) that participants correctly disposed of compostable packaging between pre- (*M* = 7.40, *SD* = 3.19) and post-intervention (*M* = 9.48, *SD* = 3.07; mean difference = 2.07, 95% CI [1.386, 2.761], *p* < .001), and between the pre-intervention phase and the follow-up phase (*M* = 9.89, *SD* = 3.08; mean difference = 2.47, 95% CI [1.779, 3.193], *p* < .001). There was no significant difference between the post-intervention and the follow-up phase (mean difference = .41, 95% CI [-.001, .826], *p* = .051).

A one-way ANOVA was also conducted to examine whether there were any changes in how confident participants were in their choices. The effect of time was statistically significant, *F*(2, 212) = 22.44, *p* < .001; partial η^2^ = .175. Bonferroni-corrected follow-up analyses highlighted that there were significant increases in self-reported confidence in choices between the pre- (*M* = 7.75, *SD* = 1.58) and post-intervention (*M* = 8.29, *SD* = 1.44; mean difference = .54, 95% CI [.244, .833], *p* < .001) and between the pre-intervention and follow-up phase (*M* = 8.52, *SD* = 1.40; mean difference = .77, 95% CI [.449, 1.088], *p* < .001). There was no significant difference between the post-intervention and follow-up phase (mean difference = .23, 95% CI [-.010, .470], *p* = .065).

Taken together, the data suggest that over the course of the intervention, participants correctly selected to put more compostable packaging in the food and garden waste bin and their confidence in their choices increased.

1. The pre-registered protocol also planned to recruit participants via the local authority’s website, however, due to time pressures and high response rates to social media advertisements, only social media was used as a recruitment strategy. [↑](#footnote-ref-0)
2. While the work primarily planned to apply the COM-B model, integration of the EAST framework arose due to project partner’s chosen application of this theory to generate intervention ideas prior to the first team meeting. [↑](#footnote-ref-1)
3. A food caddy is a small container that is designed to be kept in the kitchen (e.g., on the countertop or in a cupboard) for collecting food waste. [↑](#footnote-ref-2)
