# Supplementary figures and images for: Development and evaluation of an intervention to increase the collection of compostable packaging from households for industrial composting
Source: Waste Manag Res. 2025 Apr 21;43(10):1636–49. doi: 10.1177/0734242X251328964 (PMC12476470; doi:10.1177/0734242X251328964)

**Figure S1.***The label applied to the front of the compostable packaging.*


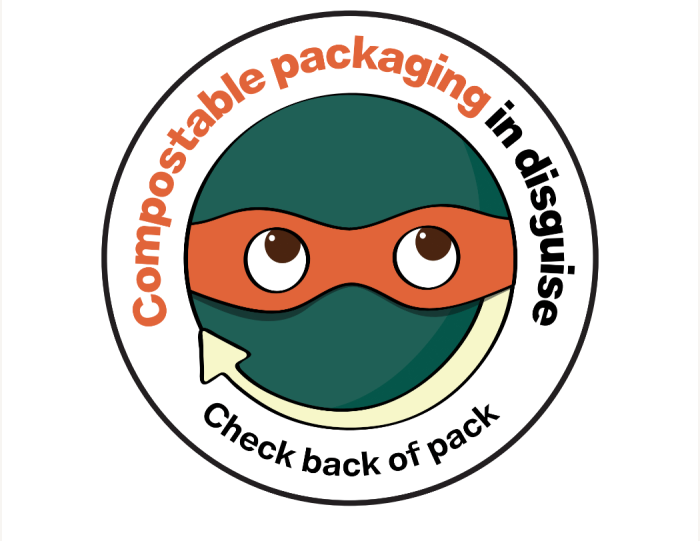

Supplement: sj-docx-2-wmr-10.1177_0734242X251328964 – Supplemental material for Development and evaluation of an intervention to increase the collection of compostable packaging from households for industrial composting [file sj-docx-2-wmr-10.1177_0734242X251328964.docx]

**Figure S2.** *The back-of-pack label applied to compostable packaging.*


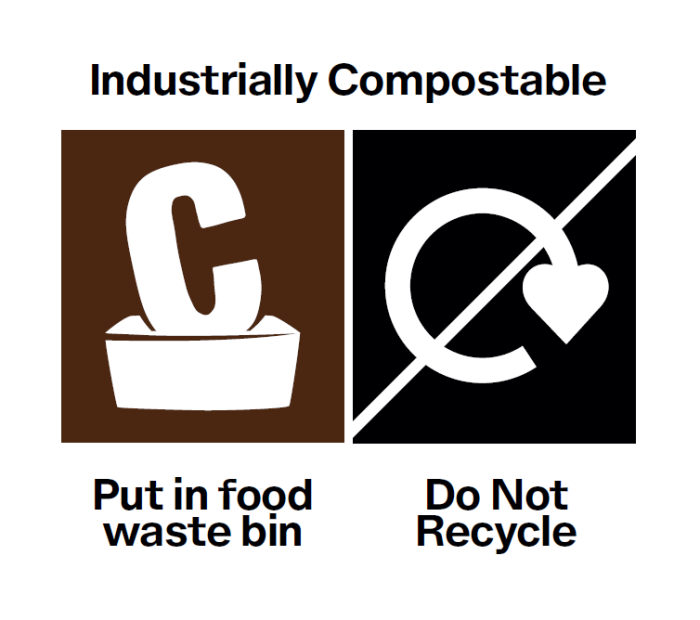

Supplement: sj-docx-3-wmr-10.1177_0734242X251328964 – Supplemental material for Development and evaluation of an intervention to increase the collection of compostable packaging from households for industrial composting [file sj-docx-3-wmr-10.1177_0734242X251328964.docx]

**Figure S3.***The tips for positioning the food caddy and food and garden waste bins.*


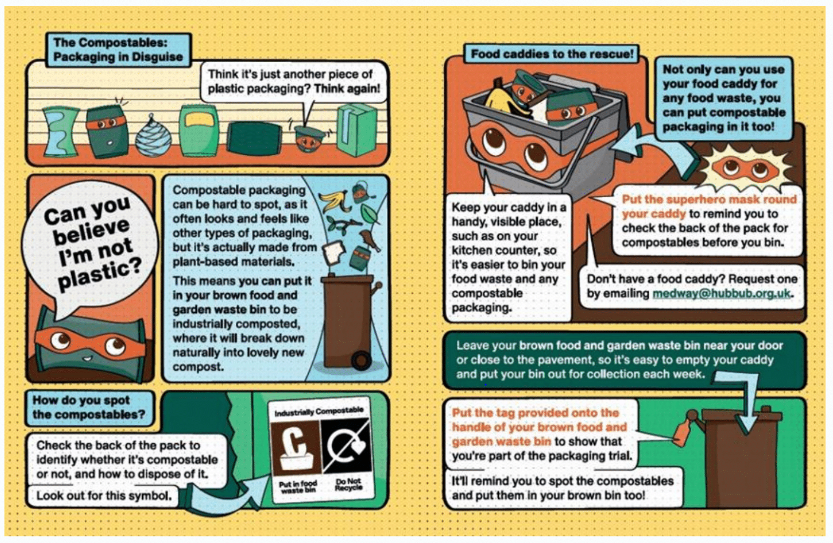

Supplement: sj-docx-4-wmr-10.1177_0734242X251328964 – Supplemental material for Development and evaluation of an intervention to increase the collection of compostable packaging from households for industrial composting [file sj-docx-4-wmr-10.1177_0734242X251328964.docx]
