# Supplementary material for: Development and evaluation of an intervention to increase the collection of compostable packaging from households for industrial composting
Source: Waste Manag Res. 2025 Apr 21;43(10):1636–49. doi: 10.1177/0734242X251328964 (PMC12476470; doi:10.1177/0734242X251328964)

**Figure S4.**

*a) Wrap for households to attach to their kitchen food caddy*

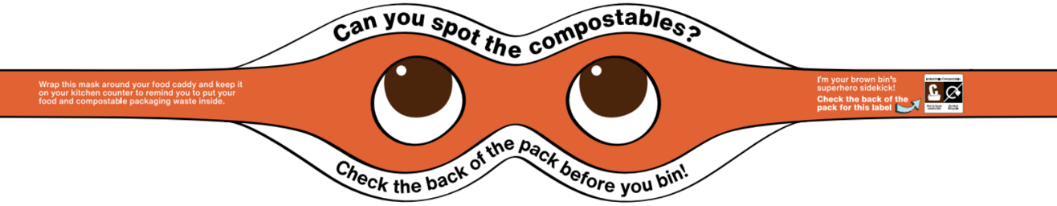


*(b) Tag for households to attach to the food and garden waste bin.*
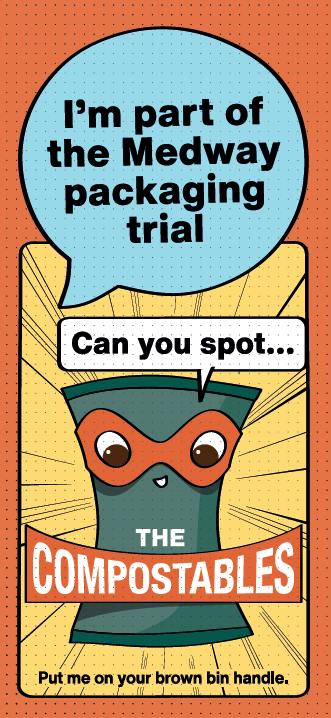

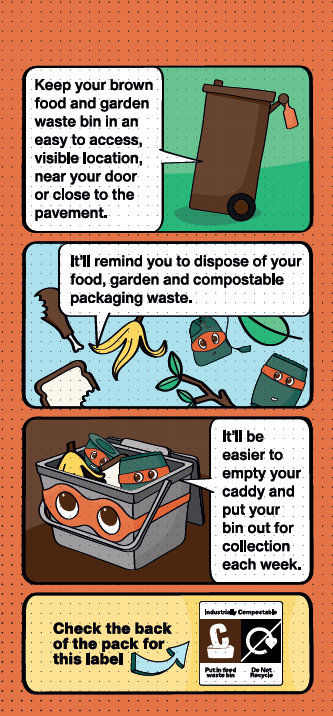

Supplement: sj-docx-5-wmr-10.1177_0734242X251328964 – Supplemental material for Development and evaluation of an intervention to increase the collection of compostable packaging from households for industrial composting [file sj-docx-5-wmr-10.1177_0734242X251328964.docx]
